# Mitochondria-targeted vitamin E analogs inhibit breast cancer cell energy metabolism and promote cell death

**DOI:** 10.1186/1471-2407-13-285

**Published:** 2013-06-13

**Authors:** Gang Cheng, Jacek Zielonka, Donna M McAllister, A Craig Mackinnon, Joy Joseph, Michael B Dwinell, Balaraman Kalyanaraman

**Affiliations:** 1 Free Radical Research Center and Department of Biophysics, Medical College of Wisconsin, Milwaukee, WI, USA; 2 Department of Pathology, Medical College of Wisconsin, Milwaukee, WI, USA; 3 Department of Microbiology and Molecular Genetics, Medical College of Wisconsin, Milwaukee, WI, USA

**Keywords:** Breast cancer metabolism, Mitochondria, Bioenergetics, Tocopherol, Antiglycolytics, Mitochondria-targeted drugs, Triphenylphosphonium cations

## Abstract

**Background:**

Recent research has revealed that targeting mitochondrial bioenergetic metabolism is a promising chemotherapeutic strategy. Key to successful implementation of this chemotherapeutic strategy is the use of new and improved mitochondria-targeted cationic agents that selectively inhibit energy metabolism in breast cancer cells, while exerting little or no long-term cytotoxic effect in normal cells.

**Methods:**

In this study, we investigated the cytotoxicity and alterations in bioenergetic metabolism induced by mitochondria-targeted vitamin E analog (Mito-chromanol, Mito-ChM) and its acetylated ester analog (Mito-ChMAc). Assays of cell death, colony formation, mitochondrial bioenergetic function, intracellular ATP levels, intracellular and tissue concentrations of tested compounds, and *in vivo* tumor growth were performed.

**Results:**

Both Mito-ChM and Mito-ChMAc selectively depleted intracellular ATP and caused prolonged inhibition of ATP-linked oxygen consumption rate in breast cancer cells, but not in non-cancerous cells. These effects were significantly augmented by inhibition of glycolysis. Mito-ChM and Mito-ChMAc exhibited anti-proliferative effects and cytotoxicity in several breast cancer cells with different genetic background. Furthermore, Mito-ChM selectively accumulated in tumor tissue and inhibited tumor growth in a xenograft model of human breast cancer.

**Conclusions:**

We conclude that mitochondria-targeted small molecular weight chromanols exhibit selective anti-proliferative effects and cytotoxicity in multiple breast cancer cells, and that esterification of the hydroxyl group in mito-chromanols is not a critical requirement for its anti-proliferative and cytotoxic effect.

## Background

Emerging research in cancer therapy is focused on exploiting the biochemical differences between cancer cell and normal cell metabolism [1,2]. A major metabolic reprogramming change that occurs in most malignant cancer cells is the shift in energy metabolism from oxidative phosphorylation to aerobic glycolysis (the Warburg effect) [3]. Strategies to selectively deplete ATP levels in tumor cells include mitochondrial targeting of lipophilic, delocalized cationic drugs [4]. Enhanced accumulation of cationic drugs in tumor mitochondria has been attributed to a higher (more negative inside) mitochondrial transmembrane potential as compared to normal cells [5].

The current chemotherapies are often associated with significant morbidity and enhanced toxic side effects. Many of the chemotherapeutic drugs are potently cytotoxic to neoplastic and normal cells, although newer targeted therapies developed against specific cancer phenotypes may potentially increase efficacy and decrease toxic side effects [6]. A major objective in cancer chemotherapy is to enhance tumor cell cytotoxicity without exerting undue cytotoxicity in normal cells. Ongoing efforts in our and other laboratories include development of cationic drugs containing triphenylphosphonium cation (TPP^+^) moiety or TPP^+^ conjugated to a naturally occurring compound (e.g., Mito-Q wherein TPP^+^ is conjugated to Co-Q) that preferentially target tumor cell mitochondria [4,7,8].

Chromanols are a family of phenolic compounds containing a chromanol ring system and an aliphatic side-chain. Tocopherols (T) and tocotrienols (TT), a group of structurally related isomeric compounds (α-, β-, γ- and δ-T and TT) consist of a chromanol ring and a 16-carbon side chain. A few of these compounds (α-T or Vit-E, γ-T and γ-TT) are present in the human diet. Isomers of T and TT exhibit cancer preventive, anti-proliferative and pro-apoptotic antitumor activity differently in xenograft tumor models [9-11]. The exact mechanisms by which these agents inhibit tumorigenesis and tumor progression remain unknown; however, various models have been put forth, ranging from their antioxidant and anti-inflammatory effects to altered redox-signaling [12,13]. Mito-chromanol (Mito-ChM) and Mito-chromanol acetate (Mito-ChMAc) are synthetic compounds containing a naturally occurring chromanol ring system conjugated to an alkyl TPP^+^*via* a side chain carbon-carbon linker sequence (Additional file 1: Figure S1). Mito-chromanol (Mito-ChM) was prepared by hydrolyzing Mito-chromanol acetate (Mito-ChMAc) (Additional file 1: Figure S1).

Recently, investigators employed a series of “redox-silent” vitamin-E analogs with the phenolic hydroxyl group replaced by a succinate moiety (α-tocopheryl succinate; α-TOS and mito-α-tocopheryl succinate, Mito-VES) and showed their antiproliferative effects in cancer cells [14,15]. Using spin-trapping measurements, increased levels of hydroxyl radical spin adducts were detected in cancer cells treated with these esterified analogs [14]. The investigators concluded that succinylation of the hydroxyl group was responsible for enhanced formation of reactive oxygen species (ROS) and cytotoxicity in cancer cells treated with α-TOS and Mito-VES [14-16]. However, it remained unclear whether modification of the phenolic hydroxyl group is a critical requirement for the observed antitumor potential of these agents. As part of our continuing efforts to understand the chemotherapeutic mechanism of mitochondria-targeted cationic drugs, we decided to reinvestigate this problem because of the potential significance of mitochondria-targeting small molecules in cancer therapy [17].

To our knowledge, there exists very little information pertaining to alteration in metabolism or bioenergetics in tumor cells treated with chromanols, mitochondria-targeted chromanols or analogs. As chromanols are active components of naturally occurring antioxidants (e.g., Vitamin-E and tocotrienols), we surmised that it is critically important to understand the changes in breast cancer cell energy metabolism induced by mitochondria targeted chromanols (Additional file 1: Figure S1). Here we report that mitochondria-targeted small-molecular weight chromanol and its acetate ester analog (Mito-ChM and Mito-ChMAc in Additional file 1: Figure S1) selectively promote cell death in nine breast cancer cell lines, but spares non-tumorigenic breast epithelial MCF-10A cells. Mito-ChM decreases intracellular ATP and inhibits proliferation of breast cancer cells. These effects are synergistically augmented by the anti-glycolytic agent 2-deoxyglucose (2-DG).

## Methods

### Chemicals

Mito-chromanol (Mito-ChM) and Mito-chromanol acetate (Mito-ChMAc) were synthesized using a modification of previously published procedures [18] (see Additional file 1: Figure S1 for chemical structures and Additional file 2: Supplementary methods). 2-deoxyglucose (2-DG), methyl triphenylphosphonium (Me-TPP^+^) and α-tocopherol (α-Toc) were purchased from Sigma-Aldrich. D-luciferin sodium salt was obtained from Caliper Life Sciences, Inc.

### Cell culture

The breast cancer cell lines MCF-7 [estrogen receptor positive (ER^+^) and human epidermal growth factor receptor 2 negative (HER2^-^)], T47D (ER^+^ and HER2^-^), MDA-MB-231 (ER^−^, and HER2^-^), SK-BR-3 (ER^-^ and HER2^+^), MDA- MB-453 (ER^-^ and HER2^+^) and MCF-10A (ER^-^ and HER2^-^) [19] were acquired in the last three years from the American Type Culture Collection, where they are regularly authenticated. MDA-MB-231-Brain (brain-seeking) were acquired in the last two years from the National Cancer Institute, where they are regularly authenticated [MDA-MB-231-Brain cells were obtained from the NCI (Dr. Patricia Steeg) and were originally from Dr. Yoneda at UTSW]. Tissue specific, MDA-MB-231-Bone (bone-seeking) and MDA-MB-231-Lung (lung-seeking) cells were the kind gift of Dr. Massague (Memorial Sloan Kettering, New York, NY) as defined previously [20,21]. Cells were stored in liquid nitrogen and used within six months after thawing. Cell lines were grown at 37°C in 5% CO_2_. MCF-7 cells were maintained in MEM-α (Invitrogen) containing 10% fetal bovine serum, bovine insulin (10 μg/ml), penicillin (100 U/ml) and streptomycin (100 μg/ml). MCF-10A cells were cultured in DMEM/F12 media (1:1) (Invitrogen) supplemented with 5% horse serum, bovine insulin (10 μg/ml), epidermal growth factor (20 ng/ml), cholera toxin (100 ng/ml), and hydrocortisone (0.5 μg/ml), penicillin (100 U/ml) and streptomycin (100 μg/ml). MDA-MB-453, MDA-MB-231, MDA-MB-231-Brain, MDA-MB-231-Bone and MDA-MB-231-Lung cells were cultured in DMEM, 10% fetal bovine serum, penicillin (100 U/ml) and streptomycin (100 μg/ml). T47D cells were cultured in RPMI 1640, 10% fetal bovine serum, penicillin (100 U/ml) and streptomycin (100 μg/ml). SK-BR-3 cells were cultured in McCoys 5A, 10% fetal bovine serum, penicillin (100 U/ml) and streptomycin (100 μg/ml). The MDA-MB-231-luc cell line stably transfected with luciferase was cultured under the same conditions as the MDA-MB-231 cells described above and were recently described in detail [22]. They were regularly assessed for standard growth characteristics, and tumorigenicity in nude mice.

### Cell death and clonogenic assays

Breast cancer cells and MCF-10A cells seeded at 1 × 10^4^ per well in 96-well plates were treated with Mito-ChM or Mito-ChMAc for 24 h, and dead cells were monitored in the presence of 200 nM Sytox Green (Invitrogen). The Sytox method labels the nuclei of dead cells yielding green fluorescence. Fluorescence intensities from the dead cells in 96-well plate were acquired in real time every 5 min for first 4 h, then every 15 min after 4 h using a plate reader (BMG Labtech, Inc.) equipped with atmosphere controller set at 37°C and 5% CO2:95% air using a fluorescence detection with 485 nm excitation and 535 nm emission. To measure the total cell number, all of the samples in each treatment group were permeabilized by adding Triton X 100 (0.065%) in the presence of Sytox Green for 3 h, and maximal fluorescence intensities were taken as 100%. Data are represented as a percentage of dead cells after normalization to total cell number for each group.

The IncuCyte™ Live-Cell Imaging system was used for kinetic monitoring of cytotoxicity as determined by Sytox Green staining at regular cell culture condition [23]. Additionally, phase-contrast and fluorescent images were automatically collected for each time point to determine morphological cell changes.

For clonogenic assay, MCF-7, MDA-MB-231 and MCF-10A cells were seeded at 300 cells per dish in 6 cm diameter cell culture dishes and treated with Mito-ChM for 4 h. After 7–14 days, the number of colonies formed was determined. The cell survival fractions were calculated according to a published protocol [24].

### Extracellular flux assay

To determine the mitochondrial and glycolytic function of MCF-7 and MCF-10A cells treated with Mito-ChM, we used the bioenergetic function assay previously described [4]. After seeding and treatment as indicated, MCF-7 cells and MCF-10A cells were washed with complete media and either assayed immediately, or returned to a CO_2_ incubator for 24, 48 or 72 h. The cells were then washed with unbuffered media as previously described [4]. Five baseline oxygen consumption rate (OCR) and extracellular acidification rate (ECAR) measurements were then recorded before injecting oligomycin (1 μg/ml) to inhibit ATP synthase, 2,4-dinitrophenol (DNP, 50 μM) to uncouple the mitochondria and yield maximal OCR, and rotenone (Rot, 1 μM) and antimycin A (AA, 10 μM) to prevent mitochondrial oxygen consumption through inhibition of Complex I and Complex III, respectively. From these measurements, indices of mitochondrial function were determined as previously described [25,26].

### Intracellular ATP measurements

After seeding and treatment as indicated, MCF-7, MDA-MB-231, and MCF-10A cells were washed with complete media and either assayed immediately, or returned to a CO_2_ incubator for 24, 48 or 72 h. Intracellular ATP levels were determined in cell lysates using a luciferase-based assay per manufacturer’s instructions (Sigma Aldrich). Results were normalized to the total protein level in cell lysate, as determined by the Bradford method (Bio-Rad).

### Measurement of intracellular concentrations of Mito-ChM and Mito-ChMAc

After incubation, cells were washed twice with ice-cold DPBS and harvested. The cell pellet was immediately frozen in liquid nitrogen and stored at −80°C. For the extraction, the pellet was homogenized in DPBS and extracted twice with dichloromethane:methanol (2:1) mixture containing 2 mM butylated hydroxytoluene (BHT) to prevent oxidation of the chromanol ring. The organic layers were combined and dried using SpeedVac. The dry residue was dissolved in ice-cold methanol containing 2 mM BHT and taken for HPLC analysis. A similar protocol was used for extraction of Mito-ChM from tissue samples from the *in vivo* xenograft experiments, but tissue homogenization and extraction were performed with the use of Omni Bead Ruptor 24 homogenizer (Omni International).

HPLC with electrochemical detection was used to detect and quantify Mito-ChM and α-tocopherol. The HPLC system (ESA) and was equipped with CoulArray detector containing eight coulometric cells connected in a series. Analytes were separated on a Synergi Polar RP column (Phenomenex, 250 mm × 4.6 mm, 4 μm) using a mobile phase containing 25 mM lithium acetate (pH 4.7) in 95% methanol. The isocratic elution with the flow rate of 1.3 ml/min was used. The voltages applied to the coulometric cells were as follows: 0, 200, 300, 600, 650, 700, 750 and 800 mV. At concentrations 10 μM and lower, the dominant peak was observed at 300 mV; at higher concentrations the dominant peak was observed at 600 mV. For quantitative analyses, the areas of peaks detected at potentials 200 – 650 mV were added and the sum was used for determining the concentration.

The simultaneous quantification of Mito-ChM and Mito-ChMAc in the extracts was performed using the UHPLC system (Shimadzu Nexera) coupled to an MS/MS detector (Shimadzu 8030). The following parameters of the MS detector were used: ionization mode: electrospray (ESI); nebulizing gas (N_2_) flow: 2 l/min; drying gas (N_2_) flow: 15 l/min; desolvation line temperature: 250°C; heat block temperature: 400°C; collision gas: Ar. The compounds were separated on a Kinetex PhenylHexyl column (Phenomenex, 50 mm × 2.1 mm, 1.7 μm) thermostated at 40°C, using a mobile phase containing 0.1% formic acid in water/acetonitrile mixture with a gradient of acetonitrile from 50% to 80% over 6 min. The flow rate was set at 0.4 ml/min. The detector was set to continuously scan the eluate in the positive mode in the m/z range between 10 and 1000. Additionally, for selective monitoring of Mito-ChM and Mito-ChMAc, the multiple reaction monitoring (MRM) transitions of 679.1 → 515.0 (for Mito-ChM) and 721.1 → 415.0 (for Mito-ChMAc) were used and the corresponding peak areas were used for quantitative analysis.

### Xenograft experiments

All protocols were approved by the Medical College of Wisconsin Institutional Animal Care and Use Committee. MDA-MB-231-luc cells (5 × 10^5^ cells in 200 μl of a mixture of 1:1 PBS/Matrigel (BD Biosciences) were injected into the right mammary fat-pad of 8-week-old female SHO mice (Charles Rivers). Tumor establishment and growth were monitored 18–24 h after receiving Mito-ChM by injecting D-luciferin as per manufacturer’s instructions (Caliper Life Sciences) and detecting bioluminescence using the Lumina IVIS-100 *In Vivo* Imaging System (Xenogen Corp.) [22]. The light intensities emitted from regions of interest were expressed as total flux (photons/second). Two days after injecting the cells, mice were imaged to verify tumor establishment. Mice were then orally gavaged with either water (control, shared group as in Reference [4]) or Mito-ChM (60 mg/kg) five times/wk (Monday through Friday). After 4 weeks of treatment and 48 h after receiving last administration the mice were sacrificed, and the tumor, kidney, heart and liver were removed. Half of tissue samples were snap-frozen in liquid nitrogen and stored at −80°C for Mito-ChM extraction, and the other half was formalin fixed and paraffin embedded for hematoxylin and eosin (H&E) staining.

### Statistics

All results are expressed as mean±SEM. Comparisons among groups of data were made using a one-way ANOVA with Tukey *post hoc* analysis. P value of less than 0.05 was considered to be statistically significant.

## Results

### Cytotoxic and anti-proliferative effects of Mito-ChM and Mito-ChMAc in breast cancer and non-cancerous cells

**Figure 1:**
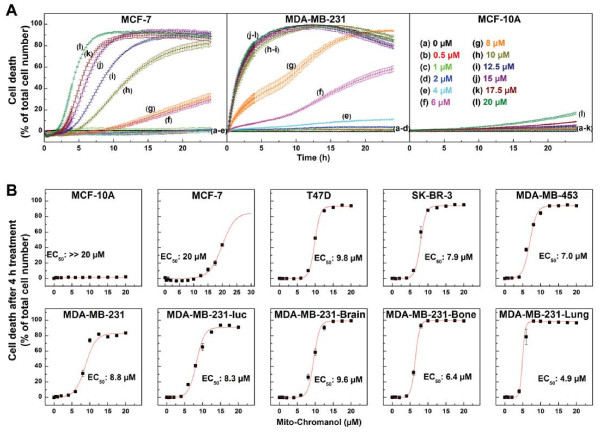
**The cytotoxic effect of Mito-ChM in breast cancer and non-cancerous cells.** Nine different breast cancer cells and MCF-10A cells were treated with Mito-ChM at the indicated concentrations (0.5-20 μM) for 24 h, and cell death was monitored in real time by Sytox Green staining. Data shown are the means ± SEM for n = 4. Real time cell death curves were plotted in panel **A** for MCF-7 (*left*), MDA-MB-231 (*middle*) and MCF-10A cells (*right*). Panel **B** shows the titration of breast cancer and non-cancerous cells with Mito-ChM, and the extent of cell death observed after 4 h treatment is plotted against Mito-ChM concentration. Solid lines represent the fitting curves used for determination of the EC_50_ values, indicated in each panel.

The dose-dependent cytotoxicity of Mito-ChM or Mito-ChMAc in nine breast cancer and non-cancerous MCF-10A cells was monitored for 24 h (Figure 1). Both Mito-ChM and Mito-ChMAc caused a dramatic increase in cytotoxicity in all nine breast cancer cell lines tested (Figure 1 and Additional file 1: Figure S2) but not in MCF-10A cells (Figure 1A and Additional file 1: Figure S2). The EC_50_ values (concentration inducing 50% of cell death) for Mito-ChM after a 4 h treatment in all cell lines tested are shown in Figure 1B. In eight out of nine breast cancer cell lines, the EC_50_ values measured for Mito-ChM were below 10 μM. The acetate ester of Mito-ChM exhibited similar but slightly higher EC_50_ values, as shown in Additional file 1: Figure S2B. With MCF-7 cells, the estimated EC_50_ for Mito-ChM at 4 h was 20 μM, while in MCF-10A we did not observe any toxicity under these conditions. The relatively higher EC_50_ value in MCF-7 cells can be ra tionalized by a delayed response to Mito-ChM, as shown in Figure 1A. Notably, the EC_50_ values of Mito-ChM in MCF-7 cells measured to be *ca*. 10.4 ± 0.2 μM and 7.8 ± 0.4 μM for a 12 and 24 h incubation period, respectively. The EC_50_ values for Mito-ChMAc under the same conditions were 11.9 ± 0.4 μM (12 h) and 8.8 ± 0.1 μM (24 h) (Additional file 1: Figure S2). In contrast, the EC_50_ values for these agents in MCF-10A cells were much greater than 20 μM (Figure 1A and Additional file 1: Figure S2) even after a 24 h incubation.

We further confirmed these results by monitoring in real time the cytotoxicity of Mito-ChM using IncuCyte (Additional file 1: Figure S3) which enabled continuous monitoring of Sytox fluorescence intensity (Additional file 3: Figure S3B) and collecting of the phase contrast and fluorescence images of the cells. The corresponding confocal fluorescence images of MCF-7 cells (marked 1–4, *top*) and MCF-10A cells (marked 5–8, *bottom*) treated with 20 μM of Mito-ChM are shown in Additional file 1: Figure S3. Results obtained using the IncuCyte are consistent with the cytotoxicity results obtained with the plate reader (Figure 1A). Notably, similar effects of Mito-ChM on cell death for 24 h treatment were observed using the endpoint Sytox Green assay, implying that incubation with Sytox probe had no adverse effect (data not shown). Incubation with α-Toc (up to 20 μM) (Additional file 1: Figure S1) in the presence and absence of Me-TPP^+^ (up to 20 μM) did not significantly increase cytotoxicity in either MCF-7 or MCF-10A cells, even after a 24 h treatment (data not shown). These results suggest that TPP^+^ conjugation to a chromanol moiety *via* the carbon-carbon linker side chain is responsible for the enhanced cytotoxic and anti-proliferative effects in breast cancer cells. These results also indicate that even the acetate ester form of Mito-ChM (*i.e.*, Mito-ChMAc) is equally cytotoxic in breast cancer cells.

**Figure 2:**
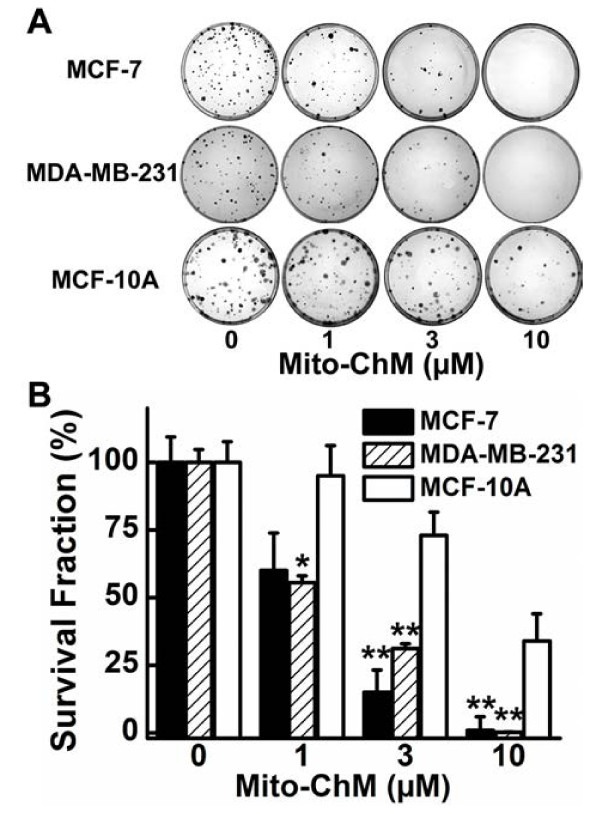
**Effects of Mito-ChM on colony formation in MCF-7, MDA-MB-231 and MCF-10A cells.** (**A**) MCF-7, MDA-MB-231 and MCF-10A cells were treated with Mito-ChM (1–10 μM) for 4 h and the colonies formed were counted. (**B**) The survival fraction was calculated under the same conditions as in (**A**). Data shown represent the mean ± SEM. *, *P* < 0.05, **, *P* < 0.01 (n = 6) comparing MCF-7 and MDA-MB-231 with MCF-10A under the same treatment conditions.

We used a clonogenic assay to monitor the anti-proliferative effects of Mito-ChM. As shown in Figure 2A, there was a dramatic decrease in colony formation in MCF-7 and MDA-MB-231 cells, as compared to MCF-10A cells, when treated with Mito-ChM (1–10 μM) for 4 h. Figure 2B shows the calculated survival fractions of MCF-7, MDA-MB-231 and MCF-10A cells. Mito-ChM significantly decreased the survival fraction in MCF-7 and MDA-MB-231 cells as compared to MCF-10A cells. Notably, the colony formation data indicate that a 4 h treatment with 3 μM Mito-ChM was sufficient to induce significant anti-proliferative effects in both MCF-7 and MDA-MB-231 cells without noticeable cell death under those conditions (Figure 1A). Taken together, we conclude that a 4 h treatment with 3 μM Mito-ChM was sufficient to inhibit cancer cell growth, without directly causing cell death at this time point.

### Effects of Mito-ChM on mitochondrial bioenergetic function in MCF-7 and MCF-10A cells

**Figure 3:**
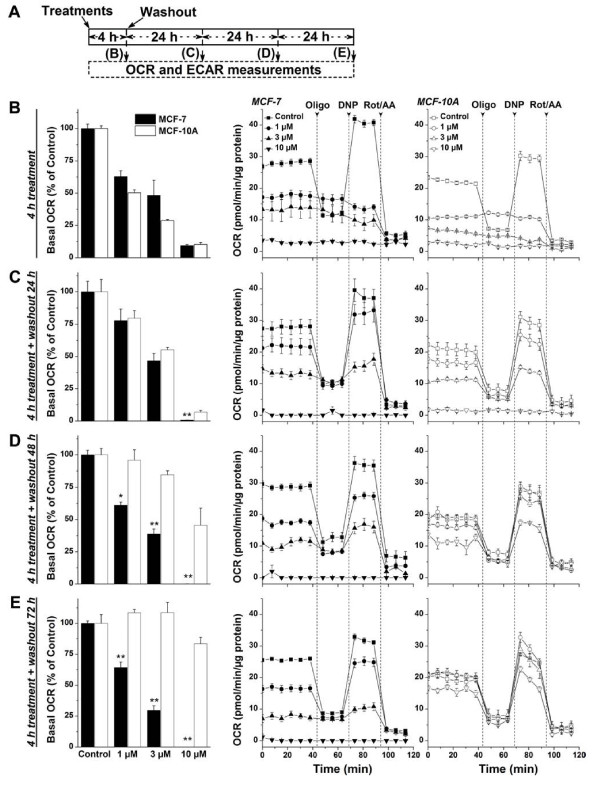
**Effects of Mito-ChM on basal OCR and bioenergetics functions in MCF-7 and MCF-10A cells.** (**A**) Experimental protocol for bioenergetic functional assay. To determine the mitochondrial and glycolytic function of MCF-7 and MCF-10A cells in response to Mito-ChM (1–10 μM), we used the bioenergetic functional assay previously described (4,25). After seeding and treatment, MCF-7 cells and MCF-10A cells were subsequently washed with complete media (MEM-α for MCF-7 and DMEM/F12 for MCF-10A) and either assayed immediately, or returned to a 37°C incubator for 24, 48, or 72 h. The relative time of treatment and post-treatment incubation that corresponds to the appropriate figures is indicated. (**B**) MCF-7 and MCF-10A cells were assayed for OCR immediately after treatment with Mito-ChM (1–10 μM) for 4 h, (**C**) after incubation without Mito-ChM for an additional 24 h, (**D**) after additional incubation without Mito-ChM for 48 h, and (**E**) after additional incubation without Mito-ChM for 72 h.

To better understand the differential cytotoxic effects of Mito-ChM, we monitored the changes in bioenergetic function with time in MCF-7 and MCF-10A cells using the XF24 extracellular flux analyzer. The experimental protocol for this experiment is shown in Figure 3A. Both cell lines were treated with Mito-ChM (1–10 μM) for 4 h, washed and returned to fresh culture media. The oxygen consumption rate (OCR) and extracellular acidification rate (ECAR) were measured immediately and after 24, 48, and 72 h (Figure 3B,C,D, and E; *left*; Additional file 3: Table S1). The effects of mitochondrial inhibitors, oligomycin (Oligo), dinitrophenol (DNP), rotenone (Rot) and antimycin A (AA) in MCF-7 and MCF-10A cells were determined (Figure 3B,C,D, and E; *right*). The use of these metabolic modulators allows determination of multiple parameters of the mitochondrial function, as described previously [4,23,24]. As can be seen, the inhibition of OCR and mitochondrial function was persistent even at 72 h after removal of Mito-ChM in MCF-7 cells, but not in MCF-10A cells (Figure 3E). The quantitative changes in bioenergetic function (ECAR, ATP-linked OCR and maximal OCR) in MCF-7 and MCF-10A cells following treatment with Mito-ChM and washout with time are shown in Additional file 3: Table S1. The striking finding is the dramatic recovery in ATP-linked OCR from Mito-ChM treatment in MCF-10A but not in MCF-7 cells at 48 to 72 h after washout (Additional file 3: Table S1). Plausible reasons for this selectivity are discussed below.

### Effects of Mito-ChM on intracellular ATP levels in MCF-7, MDA-MB-231 and MCF-10A cells

**Figure 4:**
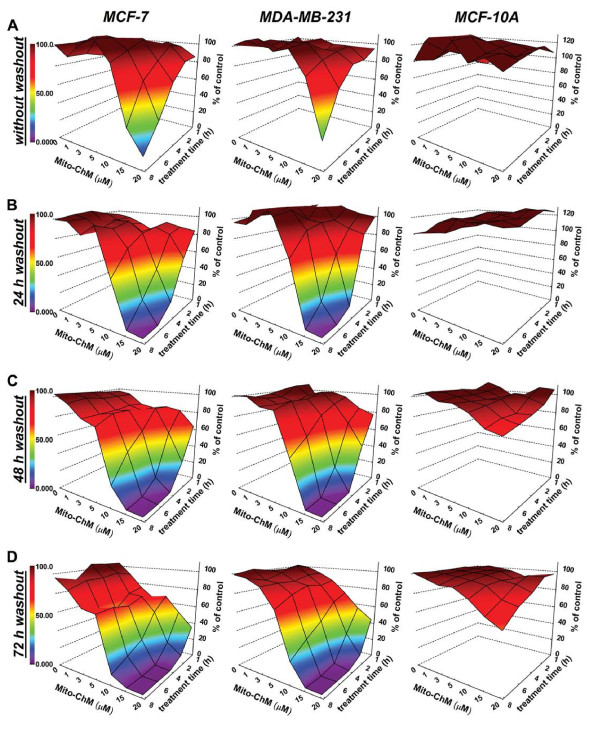
**The effect of Mito-ChM on intracellular ATP levels in MCF-7, MDA-MB-231 and MCF-10A cells.** (**A**) The MCF-7, MDA-MB-231 and MCF-10A cells seeded in 96-well plates were treated with Mito-ChM (1–20 μM) as indicated for 1–8 h. After treatment, cells were washed with complete media and either assayed immediately, or returned to cell culture incubator for (**B**) 24 h, (**C**) 48 h, or (**D**) 72 h. Intracellular ATP levels were measured using a luciferase-based assay. Data are represented as a percentage of control (non-treated) cells after normalization to total cellular protein for each well. The calculated absolute values of ATP (nmol ATP/mg protein) for MCF-7, MDA-MB-231 and MCF-10A cells are shown in Additional file 3: Tables S2, S3 and S4, respectively.

The intracellular ATP levels in MCF-7, MDA-MB-231 and MCF-10A cells treated with different concentrations of Mito-ChM (1–20 μM) for 1–8 h, immediately and after a 24–72 h washout period, were measured using a luciferase-based assay [22]. The absolute values of intracellular ATP levels (after normalization to total protein content) in MCF-7, MDA-MB-231 and MCF-10A cells following treatment with Mito-ChM are shown in Additional file 3: Tables S2, S3 and S4. Figure 4 (A-D) shows a heat map representation of intracellular ATP levels in these cells (colored areas from brown to purple indicate a progressive decrease in ATP from 100% to 0%). As shown, Mito-ChM induced a decrease in intracellular ATP levels in MCF-7 and MDA-MB-231 but not in MCF-10A cells, even after a 72 h washout in a time- and concentration-dependent manner. For example, a 4 h treatment with Mito-ChM (15 μM) followed by a 48 h washout decreased ATP (nmol ATP/mg protein) in MCF-7 cells from 22.3 ± 0.6 to 3.3 ± 0.2, in MDA-MB-231 cells from 26.0 ± 0.9 to 7.1 ± 1.3 and in MCF-10A cells from 25.6 ± 0.4 to 21.9 ± 1.2. These results suggest that Mito-ChM treatment strongly inhibits intracellular energy metabolism in MCF-7 and MDA-MB-231 but not in MCF-10A cells.

### Enhanced sequestration of Mito-ChM in MCF-7 and MDA-MB-231 cells

**Figure 5:**
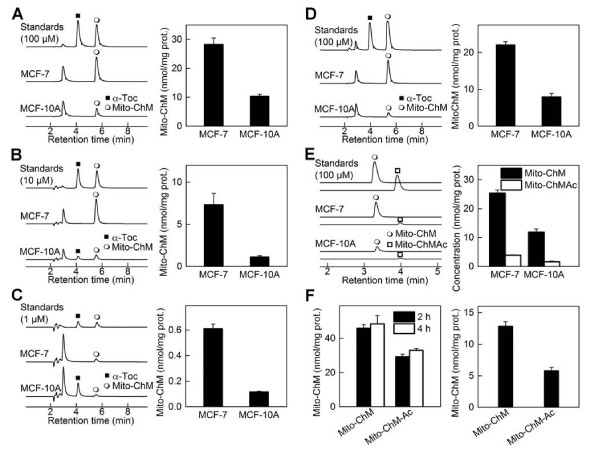
**Intracellular accumulation of Mito-ChM in MCF-7, MDA-MB-231 and MCF-10A cells.** (**A**) HPLC-EC chromatograms (dominant channels) of the mixture of standards (100 μM) of α-tocopherol and Mito-ChM, and of extracts from cells treated for 4 h with 10 μM Mito-ChM (*left panel*). Quantitative data on intracellular concentration of Mito-ChM after normalization to protein content (*right panel*). (**B**) Same as in panel **A**, but after a 4 h treatment with 10 μM Mito-ChM, medium was changed and cells incubated further for another 24 h in culture medium in the absence of Mito-ChM. Chromatogram of standards represents a mixture of α-tocopherol and Mito-ChM (10 μM each). (**C**) Same as in panel **A**, but cells were treated for 48 h with 1 μM Mito-ChM. Chromatogram of standards represents a mixture of α-tocopherol and Mito-ChM (1 μM each). (**D**) Same as in panel **A**, but cells were treated for 4 h with 10 μM Mito-ChMAc. (**E**) HPLC-MS/MS chromatograms [MRM transitions: 679.1 → 515.0 for Mito-ChM (*upper traces*) and 721.1 → 415.0 for Mito-ChMAc (*lower traces*)] of the mixture of standards (100 μM) of Mito-ChM and Mito-ChMAc, and of extracts from cells treated for 4 h with 10 μM Mito-ChMAc (*left panel*). Quantitative data on intracellular concentrations of Mito-ChM and MitoChMAc after normalization to protein content (*right panel*). (**F**) Intracellular levels of Mito-ChM in MDA-MB-231 cells incubated for 2 and 4 h with 10 μM Mito-ChM or Mito-ChMAc (*left panel*). Right panel shows similar data, but after 4 h incubation with the compounds, cells were incubated for 24 h in culture medium alone.

We used HPLC with electrochemical detection (HPLC-EC) to measure the intracellular concentrations of Mito-ChM in MCF-7, MDA-MB-231 and MCF-10A cells. Treatment of MCF-7 and MCF-10A cells with Mito-ChM for 4 h resulted in the accumulation of Mito-ChM in both cell lines, but their levels in MCF-7 cells were 2.7-fold higher than in MCF-10A cells (Figure 5A). Incubation of the same cells for an additional 24 h in Mito-ChM-free media caused a more pronounced difference in intracellular levels of Mito-ChM in MCF-7 and MCF-10A cells (Figure 5B). Incubation with 1 μM of Mito-ChM for 48 h caused a 6-fold difference in intracellular accumulation of Mito-ChM (Figure 5C). Similar experiments were performed using Mito-ChMAc. Mito-ChMAc underwent intracellular hydrolysis, forming mostly Mito-ChM in both cell lines after a 4 h incubation (Figure 5D). This was further confirmed by LC-MS/MS equipped with multiple reaction-monitoring capabilities. Incubation of both MCF-7 and MCF-10A cells with 10 μM Mito-ChMAc caused significantly higher levels of Mito-ChM (*ca*. 85% of total amount) as compared to Mito-ChMAc (*ca.* 15%), with no apparent differences in hydrolytic activities between both cell lines (Figure 5E). Consistent with Figure 5A, the intracellular concentration of Mito-ChM was significantly higher in MCF-7 cells as compared to MCF-10A following a 4 h treatment with Mito-ChMAc. Similar to MCF-7 cells, enhanced accumulation of Mito-ChM was also observed in MDA-MB-231 cells (Figure 5F).

### Effects of Mito-ChM on tumor growth: Breast cancer xenograft model

**Figure 6:**
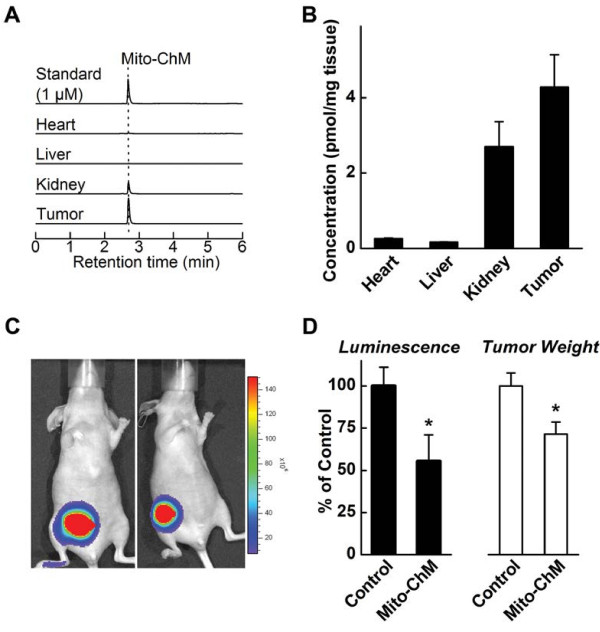
**Tissue accumulation and tumor growth inhibitory activity of Mito-ChM in *in vivo *MDA-MB-231-luc xenograft model.** (**A**) HPLC-MS/MS chromatograms (MRM transition: 679.1 → 515.0) of the Mito-ChM standard (1 μM), and of indicated tissue extracts from MDA-MB-231-luc tumor xenograft mice treated with Mito-ChM. Quantitative data on concentrations of Mito-ChM after normalization to tissue wet weight are shown in panel **B**. Tumor growth was determined by both bioluminescence signal intensity and tumor wet weight after 4 weeks of treatment. Representative bioluminescent images are show in (**C**). Quantitative data were plotted in panel **D** on bioluminescence signal intensity (*left*) and wet tumor weight (*right*). Data are represented as a percentage of control mice, mean ± SEM (n = 10, control group and n = 9, Mito-ChM treated group). *, P < 0.05 vs. control group.

We investigated the ability of Mito-ChM to exert chemotherapeutic effects in an *in vivo* breast tumor model. First, we tested the accumulation of Mito-ChM in tumor tissue, as compared with selected organs, including heart, liver and kidney (Figure 6A and B). Mito-ChM accumulated selectively in tumor and kidney, but not in heart or liver tissue, as measured 48 h after receiving the last dose of Mito-ChM. Administration of Mito-ChM led to a 45% decrease in the bioluminescence signal intensity (total flux) as compared to the control mice after 4 weeks of treatment (Figure 6C and D). Furthermore, this treatment significantly diminished tumor weight (Figure 6D) by 30% as compared to the control mice, without causing significant changes in kidney, liver and heart weights or other major morphological changes (as determined by H&E staining in Additional file 1: Figure S4 and Additional file 3: Table S5).

### Antiglycolytic agents synergistically enhance the anti-proliferative and cytotoxic effects of Mito-ChM and Mito-ChMAc

**Figure 7:**
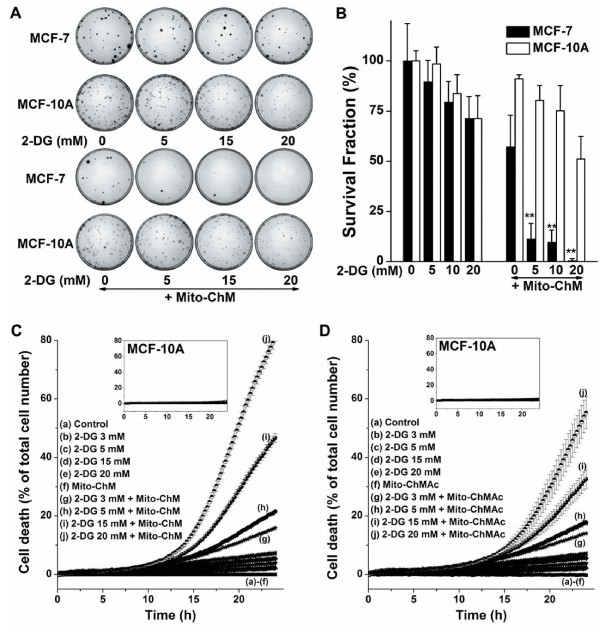
**Synergistic cytotoxicity and anti-proliferative effects of Mito-ChM and 2-DG.** (**A**) Representative pictures of the colonies formed. MCF-7 and MCF-10A cells were treated with 2-DG only (*upper*) or 2-DG in the presence of Mito-ChM (1 μM) (*lower*) for 4 h and the colonies formed were counted. (**B**) The survival fraction was calculated under the same conditions as in (**A**). (**C**,**D**) Cytotoxic effects of the combination of Mito-ChM and 2-DG. MCF-7 (main panels) and MCF-10A (inserts) cells were treated with 2-DG alone (at the indicated concentrations), 2-DG in the presence of 1 μM Mito-ChM (**C**) or 1 μM Mito-ChMAc (**D**) for 24 h and cell death was monitored in real time by Sytox Green staining. Data shown are the mean ± SEM. n = 4-6.

At higher concentrations (= 10 μM), Mito-ChM inhibits both OCR and ECAR and exerts selective toxicity to MCF-7 cells (Figure 3 and Additional file 3: Table S1). We decided to investigate whether dual targeting with mitochondrial and glycolytic inhibitors would enhance the efficacy of Mito-ChM at lower concentrations (≈ 1 μM). To this end, cells were treated with Mito-ChM combined with glycolytic inhibitor, 2-deoxyglucose (2-DG). As shown in Figure 7A, there was a substantial decrease in colony formation in MCF-7 cells when treated with 2-DG in the presence of 1 μM Mito-ChM. Mito-ChM more potently decreased the survival fraction in MCF-7 cells as compared to MCF-10A cells in the presence of 2-DG (Figure 7B). The combined treatment with 2-DG and 1 μM Mito-ChM or 1 μM Mito-ChMAc also caused a dramatic increase in cytotoxicity in MCF-7 as compared to MCF-10A cells (Figure 7C and *inset*; Figure 7D and *inset*). Live cell imaging and kinetic monitoring of cytotoxicity using the IncuCyte system also revealed similar results (Additional file 1: Figure S5).

## Discussion

In this study we report the use of relatively nontoxic cationic mitochondria-targeted synthetic compounds containing a naturally-occurring chromanol ring system to selectively inhibit breast cancer cell energy metabolism and promote anti-proliferative effects and cytotoxicity. These effects were synergistically enhanced in combination with anti-glycolytic agents (e.g.*,* 2-DG). In this study we also report that both Mito-ChM and its acetate ester analog, Mito-ChMAc, are nearly equipotent and exert selective toxicity in breast cancer cells.

### Mitochondria targeting of cationic compounds in cancer therapy

Lipophilic, delocalized cationic compounds were used to target tumor mitochondria because of a higher (more negative inside) mitochondrial transmembrane potential in tumor cells as compared to normal cells [27,28]. Rhodamine-123 (Rh-123) is a lipophilic, cationic fluorescent dye that was used as an indicator of the transmembrane potential. Rh-123 was shown to be retained longer (2–3 days) in the mitochondria of tumor-derived cells than in mitochondria of normal epithelial-derived cells [29]. The increased uptake and retention of Rh-123 in cancer cells correlated well with its selective and enhanced toxicity in cancer cells. However, Rh-123 inhibited cancer cell growth at much higher concentrations than did Mito-ChM. Rh-123 treatment alone (up to 100 μM for 6 h treatment) did not cause significant intracellular ATP depletion in MCF-7 cells; however, the combined treatment of Rh-123 (30 μM) and 2-DG induced a rapid loss of ATP in MCF-7 cells (data not shown). In contrast, Mito-ChM or Mito-ChMAc alone induced ATP depletion in MCF-7 and MDA-MB-231 cells. Interestingly, Mito-ChM did not significantly deplete intracellular ATP levels in non-cancerous MCF-10A cells, even though it inhibited mitochondrial respiration upon direct treatment (Figure 3B). This may be interpreted in terms of the differences in the potential to stimulate glycolysis (to compensate for inhibition of ATP production by mitochondrial respiration) in cancerous MCF-7 cells and non-cancerous MCF-10A cells. We have recently shown that MCF-10A cells have significantly higher glycolytic potential, as compared to MCF-7 cells [4]. Other mechanisms of selective retention of ATP upon direct treatment with Mito-ChM in non-cancerous MCF-10A cells cannot be excluded.

Selective uptake and retention of TPP^+^-based mitochondria targeted drugs in breast cancer cells is facilitated by a combination of several factors, including the lipophilicity of the delocalized cation, the ability to optimize the length of the linking carbon chain, and mitochondrial membrane potential. Mito-ChM and Mito-ChMAc are sequestered into cancer cells to a greater extent as compared to normal cells, and in tumor and kidney, as compared to heart or liver in treated mice. A major reason for this selective chemotherapeutic effect is attributed to the preferential and prolonged accumulation of these compounds in breast cancer cells. In addition, mito-chromanols are exquisitely more selective in inhibiting breast cancer cell growth as compared to other mitochondria-targeted drugs (e.g., Mito-CP and Mito-Q). Both Mito-ChM (with an antioxidant phenolic hydroxyl group intact) and Mito-ChMAc (lacking a free hydroxyl group) are equally potent in breast cancer cells. The cytotoxic activity of Mito-ChMAc may be attributed to the hydrolyzed form (Mito-ChM), as we observed significant hydrolysis of the compound both in breast cancer and non-cancerous cells. This finding calls into question the critical necessity for blocking the phenolic hydroxyl group by the succinate moiety in previous studies reporting the anticancer activity of mitochondria-targeted vitamin E succinate (Mito-VES) [15].

In this context, it is important to highlight the safety profile of Mito-Q_10_, a related mitochondria-targeted antioxidant, in animals and in humans [30]. Although, under *in vitro* conditions, this drug has been shown to generate detectable levels of reactive oxygen species, prolonged treatment with this drug did not increase oxidative damage or ROS levels *in vivo*[30]. As discussed in a recent review article [30], measurement of ROS as a minor pro-oxidant reaction *in vitro* does not mean that ROS generation from these drugs is a major mechanism of cancer cell death.

### Energy metabolism, metabolic reprogramming, and mitochondrial function in cancer therapy

Recent research revealed a regulatory link between glucose metabolism and expression of oncogenes and tumor suppressors in cancer cells [31-33]. Previous work has revealed that cancer-promoting oncogenes and hypoxia-inducible factor (HIF-1α) induce a glycolytic shift [34]. Activation of oncogenic signaling pathways involving PI3K/AkT/mTOR, c-Myc, Src, and Ras results in an enhanced glucose uptake and glycolytic activity, mimicking the Warburg phenotype in cancer cells [35,36]. Suppression of mitochondrial energy metabolism in breast cancer cells would potentially counteract the aerobic glycolysis advantage acquired through metabolic reprogramming. Targeting of both mitochondrial bioenergetic function and the glycolytic pathway is a promising chemotherapeutic strategy [31,37]. However, the key to successful cancer therapy remains to be the selectivity [4,38]. In this regard, Mito-ChM and analogs offer a unique advantage. Coupling energy restriction-mimetic agents (e.g., thiazolidinediones) with mitochondria-targeted agents may be a very effective small-molecule based anticancer therapy [39].

### Chemotherapy and ATP depletion

The present results indicate that Mito-ChM or Mito-ChMAc (1–20 μM) decreased intracellular ATP levels in a concentration- and time-dependent manner (Figure 4). The intracellular levels of Mito-ChM could decrease *via* the pumping mechanism of p-glycoprotein or MDR-1, a multidrug transporter [40]. However, as ATP is required for the pumping mechanism of p-glycoprotein, Mito-ChM-induced depletion of ATP could hinder the pump activity, thereby accumulating the cationic drug. Thus, a key advantage of using mito-chromanols in combination with a chemotherapeutic drug that induces multi-drug resistance through elevated p-glycoprotein expression might be the depletion of intracellular ATP. Intracellular ATP levels reportedly regulate chemoresistance in colon cancer cells [41]. A recent report indicates that the use of mitochondrially targeted drugs could counteract ABCA1-dependent resistance of the lung carcinoma cells [42].

It is well known that redox-based chemotherapeutics induce depletion of intracellular ATP levels *via* increased oxidative stress [43]. Unfortunately, these drugs also cause toxic side effects through oxidative mechanism of activation [44]. In this study, we show that mitochondria-targeted cationic drugs deplete intracellular ATP, not through redox activation mechanism, but through selective inhibition of ATP-linked respiration in tumor cells. As shown in Figure 4, this inhibitory effect is prolonged and permanent in breast cancer cells but not in control, noncancerous cells.

### Mitochondria-targeted cationic antioxidants: maximizing therapeutic index

The added value of mitochondria targeted cationic drugs attached to a functionally-active antioxidant group is that they will afford increased cytoprotection in normal cells through inhibition of mitochondrial oxidative damage caused by conventional chemotherapy. The therapeutic potential of mitochondria targeting in cancer therapy enhances the overall therapeutic index. Mitochondria-targeted vitamin E analog inhibits oxidative stress in normal cells [18,45]. Previous studies have shown that mitochondria-targeted drugs (Mito-Q and Mito-CP) effectively mitigated cardiotoxic and nephrotoxic side effects induced by antitumor drugs, doxorubicin and cis-platin [46,47].

Several attempts have been made to make use of the Warburg phenotypic trait (aerobic glycolysis) in cancer chemotherapy [48,49]. However, this approach has not yielded a viable chemotherapeutic strategy because of the systemic toxicity of the high concentrations of 2-DG typically used in these studies [31]. The combined inhibition of glycolysis and mitochondrial function allows the use of much lower concentrations of 2-DG. The present studies suggest that a dual targeting of relatively nontoxic mito-chromanols and glycolytic inhibitors is a viable and generalized chemotherapeutic approach. Mito-chromanols exhibit considerable tumor selectivity as evidenced by a similar EC_50_ value in eight different breast cancer cells with different genetic backgrounds. The role of stromal cells and the tumor microenvironment in general in modulating tumour sensitivity is crucial to developing successful anticancer therapeutics. Future studies should focus on investigating the effect of mitochondria-targeted small molecules on stromal cells alone and on the combination of cancer cells with stromal cells.

## Conclusion

We report a novel and selective chemotherapeutic strategy using mitochondria-targeted chromanol and its acetylated ester analog to selectively inhibit breast cancer cell energy metabolism and proliferation and promote cytotoxicity. For maximal therapeutic index, it is essential to use mitochondria-targeted, TPP^+^-conjugated cationic drug attached to a functionally active antioxidant group (nitroxide, nitrone, chromanol, or ubiquinone).

## Abbreviations

AA: Antimycin A; ATP: Adenosine-5′-triphosphate; BHT: Butylated hydroxytoluene; 2-DG: 2-deoxyglucose; DNP: 2,4-dinitrophenol; EC50: Concentration inducing 50% of cell death; ECAR: Extracellular acidification rate; ER: Estrogen receptor; HER2: Human epidermal growth factor receptor 2; H&E staining: Hematoxylin and eosin staining; HPLC-EC: HPLC with electrochemical detection; Me-TPP+: Methyl triphenylphosphonium; Mito-ChM: Mitochondria-targeted chromanol; Mito-ChMAc: Mitochondria-targeted chromanol acetylated ester analog; Mito-CP: Carboxy Proxyl conjugated to TPP^+^; Mito-Q: Co-enzyme Q conjugated to TPP^+^; Mito-VES: Mito-α-tocopheryl succinate; MRM: Multiple reaction monitoring; OCR: Oxygen consumption rate; Oligo: Oligomycin; Rh-123: Rhodamine-123; ROS: Reactive oxygen species; Rot: Rotenone; T: Tocopherols; α-Toc: α-tocopherol; α-TOS: α-tocopheryl succinate; TPP+: Triphenylphosphonium cation; TT: Tocotrienols; Vit-E: Vitamin-E.

## Competing interests

The authors declare that they have no competing interests.

## Authors’ contributions

GC and JZ worked closely at all stages of the study and conducted most of the experiments; DMM conducted the *in vivo* work. ACM carried out and analyzed the pathology work. JJ synthesized the compounds tested in the study. MBD provided some of the cell lines tested and contributed to writing the manuscript. BK and GC conceived of the study, participated in its design, and wrote the manuscript. All authors were involved in drafting, revising and approving the final manuscript.

## Pre-publication history

The pre-publication history for this paper can be accessed here:

http://www.biomedcentral.com/1471-2407/13/285/prepub

## Supplementary Material

Additional file 1: Figure S1 Chemical structures of Mito-ChM, Mito-ChMAc, α-Toc, Me-TPP^+^ and 2-deoxy-D-glucose (2-DG). **Figure S2**: The cytotoxic effect of Mito-ChMAc in breast cancer and non-cancerous cells. Nine different breast cancer cells and MCF-10A cells were treated with Mito-ChMAc, and cell death was monitored in real time by Sytox Green staining. **Figure S3**: The effect of Mito-ChM on the extent of cell death in MCF-7 and MCF-10A cells. Cell death was monitored in real time with IncuCyte by Sytox Green staining. The corresponding representative fluorescence images are shown. **Figure S4**: Hematoxylin and eosin (H&E) staining. Representative images of tissue collected from control and Mito-ChM-treated mice. **Figure S5**: Effect of 2-DG and Mito-ChM (1 μM) on the extent of cell death in MCF-7 and MCF-10A cells. Cell death was monitored in real time with IncuCyte. The corresponding representative fluorescence images are shown. **Figure S6**: Scheme of the multistep synthesis of Mito-ChMAc. Click here for file

Additional file 2 **Supplementary methods.** Supplemental text describing synthetic protocol for Mito-ChM and Mito-ChMAc. Click here for file

Additional file 3: Table S1 Effects of Mito-ChM on ECAR, ATP-linked OCR and maximal OCR in MCF-7 and MCF-10A cells. Cells were treated with Mito-ChM as indicated in Figure 3. The quantitative changes in bioenergetic functional parameters following treatment at different time periods after washout are shown. **Table S2**, **S3** and **S4**: The effect of Mito-ChM on intracellular ATP levels in MCF-7, MDA-MB-231 and MCF-10A cells, respectively. The absolute values of intracellular ATP levels (after normalization to total protein content, nmol ATP/mg protein) in MCF-7, MDA-MB-231 and MCF-10A cells following treatment with Mito-ChM are shown in **Table S2**, **S3** and **S4** while as percentage data were shown in Figure 4 as heat map figures. **Table S5**: Effects of Mito-ChM on body weight and tissue weight in xenograft mouse models. The total body weight and weights of kidney, liver and heart in control and Mito-ChM treated mice for 4 weeks are provided. Click here for file
