# Supplementary material for: Mitochondria-targeted vitamin E analogs inhibit breast cancer cell energy metabolism and promote cell death
Source: BMC Cancer. 2013 Jun 13;13:285. doi: 10.1186/1471-2407-13-285 (PMC3686663; doi:10.1186/1471-2407-13-285)
Supplement: Additional file 1: Figure S1 — Chemical structures of Mito-ChM, Mito-ChMAc, α-Toc, Me-TPP+ and 2-deoxy-D-glucose (2-DG). Figure S2: The cytotoxic effect of Mito-ChMAc in breast cancer and non-cancerous cells. Nine different breast cancer cells and MCF-10A cells were treated with Mito-ChMAc, and cell death was monitored in real time by Sytox Green staining. Figure S3: The effect of Mito-ChM on the extent of cell death in MCF-7 and MCF-10A cells. Cell death was monitored in real time with IncuCyte by Sytox Green staining. The corresponding representative fluorescence images are shown. Figure S4: Hematoxylin and eosin (H&E) staining. Representative images of tissue collected from control and Mito-ChM-treated mice. Figure S5: Effect of 2-DG and Mito-ChM (1 μM) on the extent of cell death in MCF-7 and MCF-10A cells. Cell death was monitored in real time with IncuCyte. The corresponding representative fluorescence images are shown. Figure S6: Scheme of the multistep synthesis of Mito-ChMAc. [file 1471-2407-13-285-S1.pdf]

Supplementary Figure 1

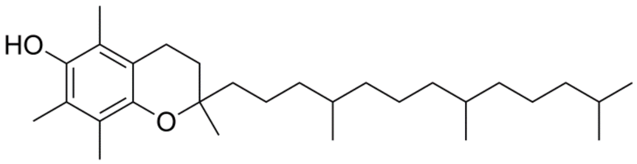

α-Toc

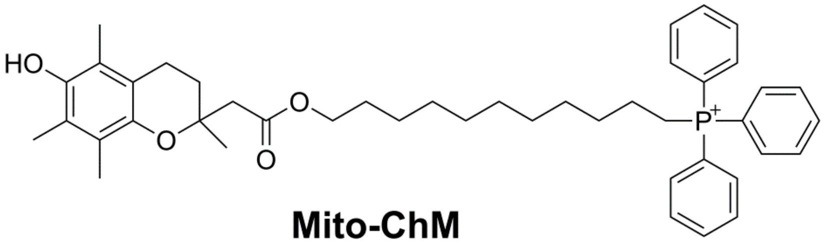

Mito-ChM

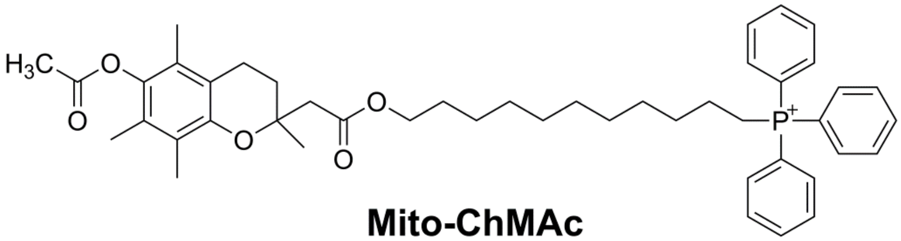

Mito-ChMAc

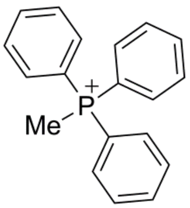

Me-TPP$^+$

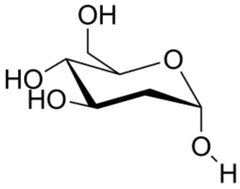

2-DG

Additional file 3, Supplementary Figure 1: Chemical structures of Mito-ChM, Mito-ChMAc, α-Toc, Me-TPP$^+$ and 2-deoxy-D-glucose (2-DG).

Supplementary Figure 2

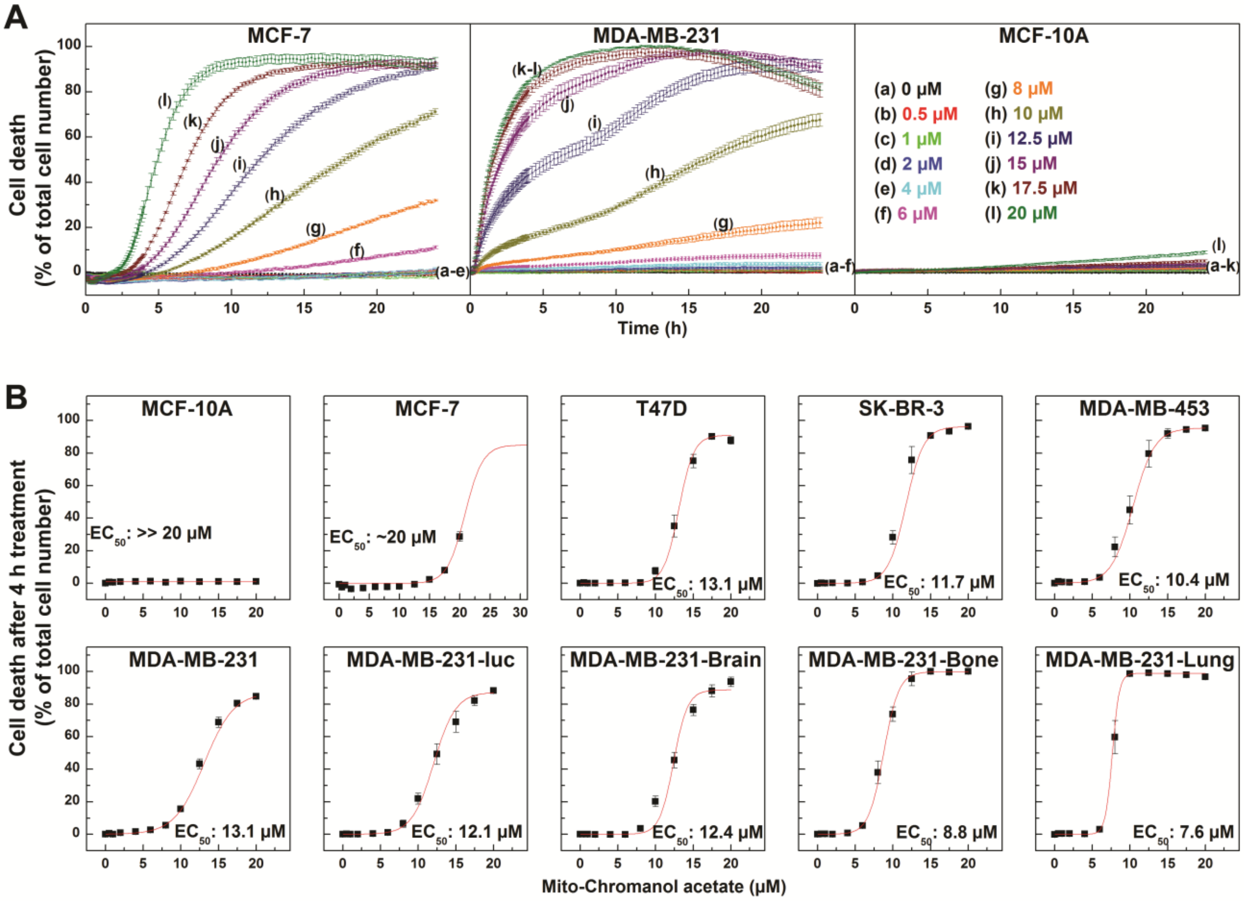

Additional file 3, Supplementary Figure 2: The cytotoxic effect of Mito-ChMAc in breast cancer and non-cancerous cells. Nine different breast cancer cells and MCF-10A cells were treated with Mito-ChMAc at the indicated concentrations (0.5-20 μM) for 24 h, and cell death was monitored in real time by Sytox Green staining. Data shown are the mean ± SEM for n=4. Real time cell death curves were plotted in panel **A** for MCF-7 (***left***), MDA-MB-231 (***middle***) and MCF-10A cells (***right***). Panel **B** shows the titration of breast cancer and non-cancerous cells with Mito-ChMAc, and the extent of cell death observed after 4 h treatment is plotted against Mito-ChMAc concentration. Solid lines represent the fitting curves used for determination of the EC_{50} values, indicated in each panel.

Supplementary Figure 3

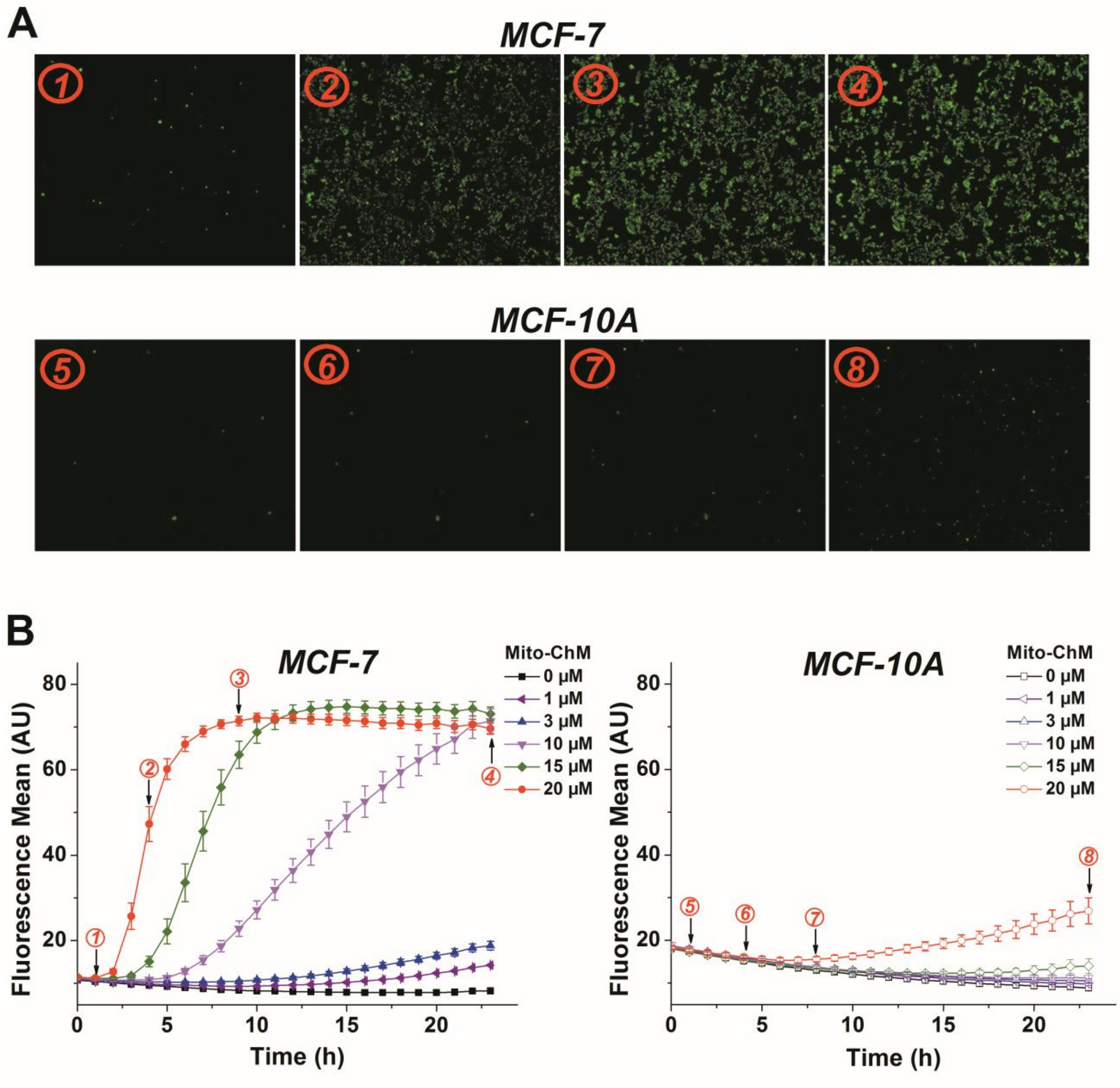

Additional file 3, Supplementary Figure 3: The effect of Mito-ChM on the extent of cell death in MCF-7 and MCF-10A cells. MCF-7 and MCF-10A cells were treated with Mito-ChM at the indicated concentrations for 23 h and cell death was monitored in real time with IncuCyte by Sytox Green staining. The corresponding representative fluorescence images are shown in panel **A** for MCF-7 cells at different time points (marked 1-4, upper) and MCF-10A cells (marked 5-8, lower) treated with 20 µM of Mito-ChM. Quantitative data are plotted in panel **B**. Data shown are the mean ± SEM, n=4.

Supplementary Figure 4

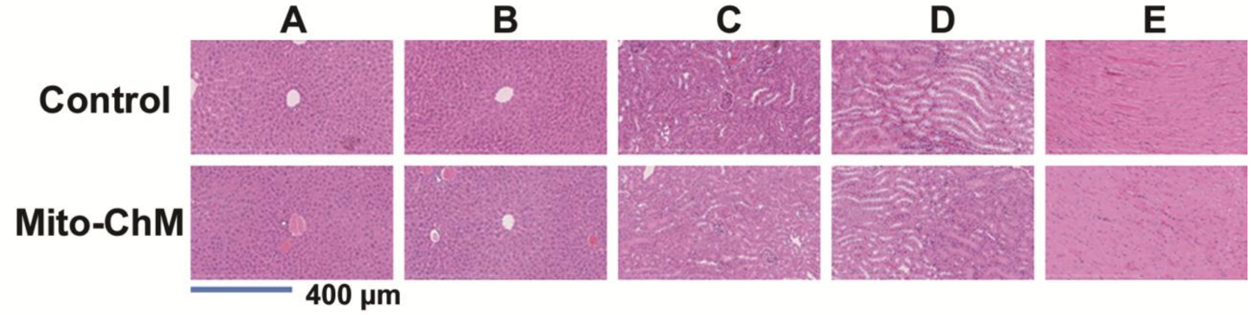

Additional file 3, Supplementary Figure 4: Hematoxylin and eosin (H&E) staining. Representative images of tissue collected from control and Mito-ChM-treated mice. Shown are examples of portal vein and hepatic duct **(A)**, central vein **(B)**, glomeruli **(C)**, renal corticomedullary junction **(D)**, and ventricle **(E)**.

Supplementary Figure 5

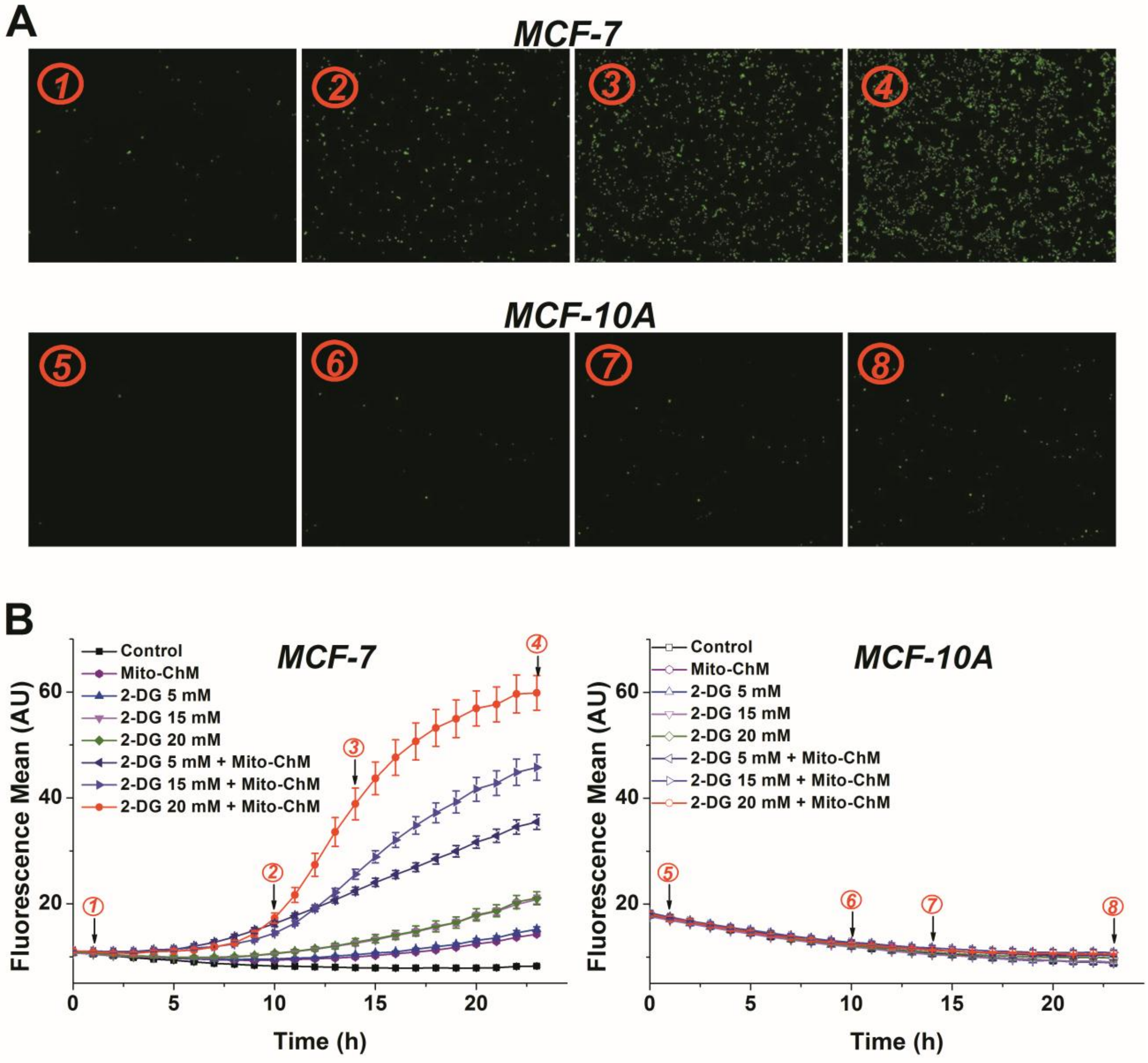

Additional file 3, Supplementary Figure 5: The effect of 2-DG and Mito-ChM (1 µM) on the extent of cell death in MCF-7 and MCF-10A cells. MCF-7 and MCF-10A cells were treated with 2-DG alone (at the indicated concentrations), and 2-DG in the presence of Mito-ChM (1 µM) and cell death was monitored in real time with IncuCyte by Sytox Green staining. The corresponding representative fluorescence images are shown in panel **A** for MCF-7 cells at different time points (marked 1-4, upper) and MCF-10A cells (marked 5-8, lower) treated with 20 mM of 2-DG in the presence of Mito-ChM. Quantitative data are plotted in panel **B**. Data shown are the mean ± SEM, n=4.

Supplementary Figure 6

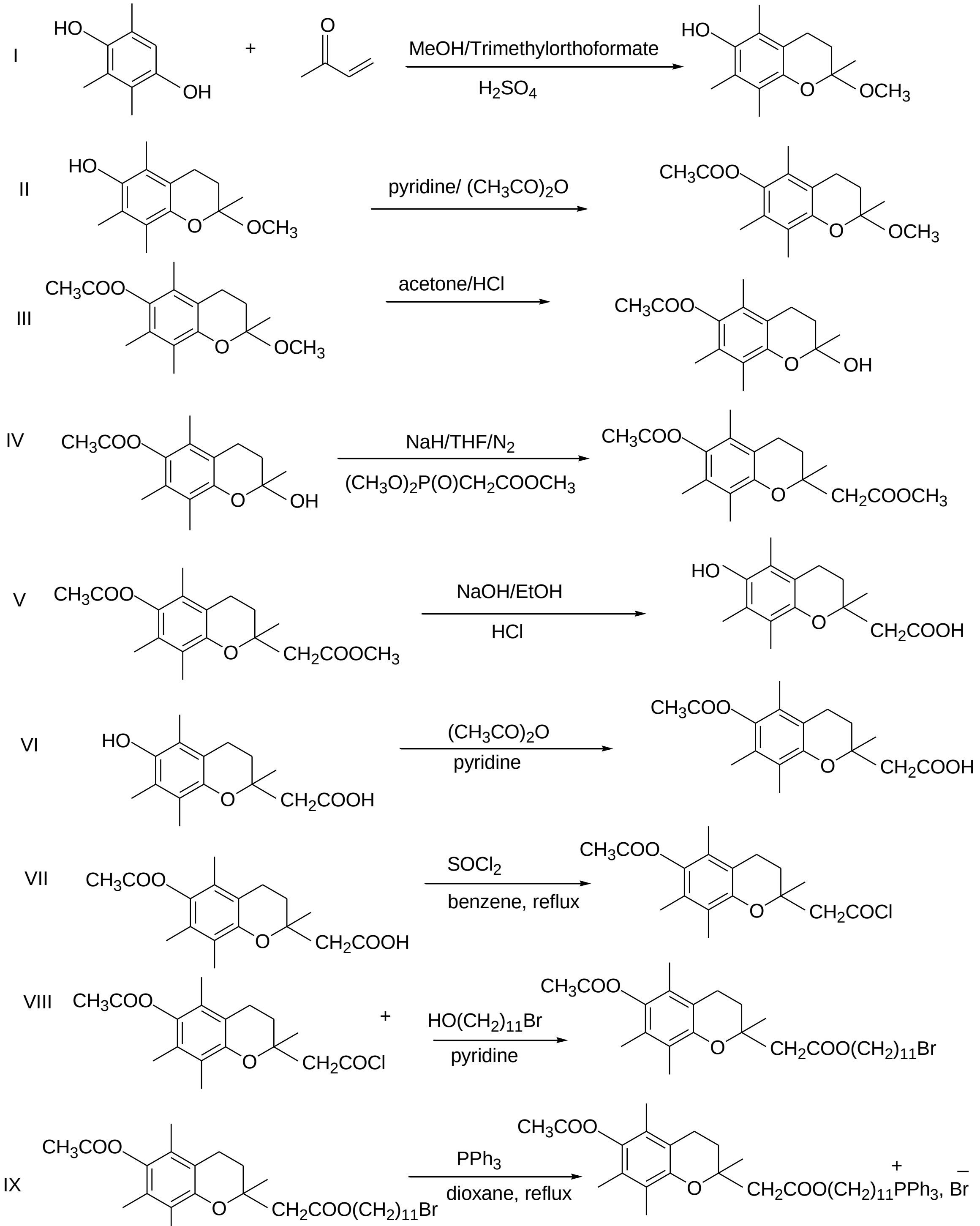

Mito-Chromanol Acetate (C11)

Additional file 3, Supplementary Figure 6: Scheme of the multistep synthesis of Mito-ChMAc.
