# Supplementary material for: Mitochondria-targeted vitamin E analogs inhibit breast cancer cell energy metabolism and promote cell death
Source: BMC Cancer. 2013 Jun 13;13:285. doi: 10.1186/1471-2407-13-285 (PMC3686663; doi:10.1186/1471-2407-13-285)
Supplement: Additional file 2 — Supplementary methods. Supplemental text describing synthetic protocol for Mito-ChM and Mito-ChMAc. [file 1471-2407-13-285-S2.pdf]

SUPPLEMENTARY METHODS

Synthetic procedure for Mito-ChMAc.

Mito-ChMAc was prepared as follows:

I. A solution of trimethylhydroquinol (0.1 mole, 15 g) in methanol (100 ml) and trimethylorthoformate (100 ml) was taken in a three-neck flask with an addition funnel and a reflux condenser and was evacuated partially and filled with nitrogen gas three times. The flask was cooled in an ice bath to about 5°C and to this cold solution, 0.25 ml of concentrated sulfuric acid was added. In the addition funnel methylvinyl ketone (0.2 mole, 17 g) was taken and added drop-wise to the hydroquinol solution in approximately two hours. After addition, the cooling bath was removed and contents kept stirred at room temperature for 36 h. The dark solution was mixed with 400 ml of ether and the organic layer was washed with water and saturated sodium bicarbonate solution. The yellow organic solution was dried over sodium sulfate and solvent removed to obtain a tan-colored solid (24 g). The impure compound was recrystallized from hot ethyl acetate to obtain 20 g of pure methoxychromanol. Thin layer chromatography (TLC) in hexane and ethyl acetate (3:1) showed a single spot. The purified compound was used in the next acetylation step.

II. To the solution of methoxychromanol (20 g) in pyridine (30 ml) was added acetic anhydride (45 ml) and kept stirred at room temperature for 20 h. Most of the liquid was removed by rotary evaporation and 200 g of ice was added to the residual liquid and stirred. The separated brown oil was extracted with ether (200 ml) and washed with cold water. The acetyl derivative was washed with HCl (0.1 M), then with saturated sodium bicarbonate solution and purified by flash-chromatography on a silica gel

column and eluted with hexane-ethyl acetate mixture (1:2). Finally, the solvent was removed to obtain a pale yellow semi-solid (25 g). TLC showed a single spot that moved ahead of the starting material. This acetate was subjected to demethylation in the following step.

III. The acetate (25 g) was dissolved in acetone (100 ml) and water (100 ml) was added, followed by concentrated HCl (7 ml). The solution was refluxed with stirring for 30 min and the acetone was slowly distilled off. After the temperature of the solution reached about 90°C, the liquid was cooled in an ice bath overnight. The separated solid was filtered and washed with water and dried. The product was recrystallized from hot ethyl acetate to get a pale yellow powder. The TLC in hexane-ethylacetate (3:1) mixture showed a single spot. The yield was 22 g.

IV. Methylacetate side-chain was introduced into the above product by reacting with trimethylphosphonoacetate in presence of sodium hydride. To a three-neck flask carrying an additional funnel was added 200 ml of tetrahydrofuran (THF), and was evacuated partially and filled with nitrogen gas three times. Under anaerobic condition, sodium hydride (0.1 mole, 4.8 g) of 50% suspension in paraffin oil was added. From the additional funnel trimethylphosphonoacetate (0.1 mole, 18 g) was added drop-wise. After addition, the solution was kept stirred for one hour at room temp. To the suspension from the additional funnel, the compound from the previous procedure (0.08 mole, 22 g) in a 50 ml THF was added drop-wise during the first one hour. The brown suspension was kept stirred at room temperature for 24 h and then refluxed for four more hours. After cooling to room temperature, water (50 ml) was added and most of the solvent was removed by rotary evaporation. Ether (200 ml) was added and the

product extracted. This was repeated with another 100 ml of ether. The combined extracts were washed with dilute HCl, dried using sodium sulfate and solvent removed to obtain the methylacetate derivative as a brown oil. This compound was further purified by flash chromatography on silica gel column and eluted with ethylacetate-hexane (1:1) solvent. TLC in the same solvent showed a single spot that moved ahead of the starting material. The product was a brown oil (26 g).

V. The ester from the above procedure was hydrolyzed by alkali. The above product was dissolved in 100 ml ethanol, and a solution of 20 g sodium hydroxide was added in 100 ml water. The solution was kept stirred at room temperature for 20 h and acidified with 5 M hydrochloric acid to about pH = 3. The light brown solid was filtered and dried in air overnight. The solid was dissolved in 100 ml of dichloromethane (DCM) and the organic layer was dried and solvent removed by rotary evaporation. The solid product was purified by flash chromatography, eluting the product with ethyl acetate. Upon solvent removal, the pure product was obtained as a dull white powder. TLC showed a single spot on development with ethylacetate. The yield of the tan powder was 22 g.

VI. The phenolic hydroxyl group was protected by acetylation. The solid was dissolved in 30 ml pyridine and 45 ml acetic anhydride was added, and the solution was stirred at room temperature for 20 h. Most of the liquid was removed by rotary evaporation and the residual semisolid was stirred in 100 ml cold water. The product was extracted twice with 50 ml DCM. The combined extract was washed with dilute HCl acid and then with sodium bicarbonate solution. The solution was dried and solvent removed to obtain a brown oil. The final product was purified by flash chromatography

and product was eluted with ethyl acetate. The TLC in ethylacetate-hexane (1:1) solvent showed a single spot. The yield was 23 g.

VII. This carboxylic acid was refluxed in 50 ml benzene containing 10 ml thionyl chloride for 1 h and solvent was removed. All the volatiles were then removed on a vacuum line.

VIII. The acid chloride was dissolved in 50 ml DCM and was slowly added to a solution of 18 g of 11-bromoundecanol and 10 ml pyridine in 100 ml DCM kept stirred in ice bath. After addition, the solution was kept stirred for an additional 20 h at room temperature. The reaction mixture was washed using 50 ml of 5 M hydrochloric acid and then with saturated sodium hydrogen carbonate solution. The solution was dried and the solvent was removed. The bromoundecanol ester was purified by chromatography after eluting with hexane and then with hexane containing increasing proportion of ether. Homogeneous fractions were combined. The TLC was performed in hexane-ethylacetate (4:1) and upon viewing by charring after sulfuric acid treatment of the plate showed a single spot. The yield of the semisolid was 30 g.

IX. Finally, the bromoundecanol ester (5.4 g, 0.01 mol) and triphenylphosphine (2.62 g, 0.01 mol) in 20 ml dioxane were heated under reflux in an oil bath kept at 90°C under nitrogen atmosphere for 48 h. The solvent was removed and 100 ml ether was added to precipitate the product as a semisolid. The impure product was purified on a silica gel chromatography and eluted with DCM and DCM containing 5% methanol. The product Mito-ChMAc was obtained as a brownish black oil. The yield was 6.5 g. The HPLC and MS analyses were performed to ascertain the purity and identity of the

product. The mass spectrometry measurement of the positive ions indicated the mass of 721, consistent with Mito-ChMAc structure.

X. Hydrolysis of Mito-ChMAc to Mito-ChM:

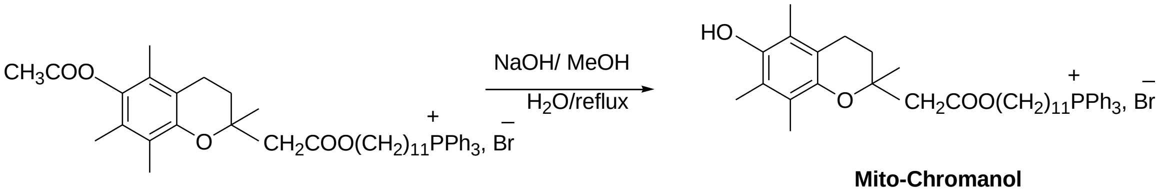

Mito-ChMAc (800 mg, 1 mmole) was dissolved in 50 ml methanol and the solution was kept under nitrogen atmosphere. To this, a solution of sodium hydroxide (40 mg, 1 mmole) in 5 ml water was added and stirred under reflux for 5 minutes. The solution was cooled and acidified with dilute HCl to pH = 6-7 and extracted with chloroform (2 x 25 ml). The organic layer was separated, dried and concentrated to a 5 ml. This was added with stirring to 50 ml ether. The precipitated semisolid was separated and purified on silicagel 60 column and eluted with 200 ml of a mixture of chloroform-ethylacetate (1:1) and the eluate was discarded. Mito-ChM was eluted with chloroform-methanol (95:5). The slow moving brown band was collected in fractions. Homogeneous fractions were combined and solvent removed to get Mito-ChM as brown semi-solid (0.5g). The HPLC and MS analyses were performed to ascertain the purity and identity of the product. The mass spectrometry measurement of the positive ions indicated the mass of 679, consistent with Mito-ChM structure.
