# Supplementary material for: Mitochondria-targeted vitamin E analogs inhibit breast cancer cell energy metabolism and promote cell death
Source: BMC Cancer. 2013 Jun 13;13:285. doi: 10.1186/1471-2407-13-285 (PMC3686663; doi:10.1186/1471-2407-13-285)
Supplement: Additional file 3: Table S1 — Effects of Mito-ChM on ECAR, ATP-linked OCR and maximal OCR in MCF-7 and MCF-10A cells. Cells were treated with Mito-ChM as indicated in Figure 3. The quantitative changes in bioenergetic functional parameters following treatment at different time periods after washout are shown. Table S2, S3 and S4: The effect of Mito-ChM on intracellular ATP levels in MCF-7, MDA-MB-231 and MCF-10A cells, respectively. The absolute values of intracellular ATP levels (after normalization to total protein content, nmol ATP/mg protein) in MCF-7, MDA-MB-231 and MCF-10A cells following treatment with Mito-ChM are shown in Table S2, S3 and S4 while as percentage data were shown in Figure 4 as heat map figures. Table S5: Effects of Mito-ChM on body weight and tissue weight in xenograft mouse models. The total body weight and weights of kidney, liver and heart in control and Mito-ChM treated mice for 4 weeks are provided. [file 1471-2407-13-285-S3.pdf]

SUPPLEMENTARY TABLES

Supplementary Table 1. Effects of Mito-ChM on ECAR, ATP-linked OCR and maximal OCR in MCF-7 and MCF-10A cells. Cells were treated with Mito-ChM (1-10 µM) as indicated in Figure 3. The quantitative changes in bioenergetic function following treatment and washout with time are shown.

Supplementary Table 2. The effect of Mito-ChM on intracellular ATP levels in MCF-7 cells. Cells were treated with Mito-ChM (1-20 µM) for indicated periods of time. After treatment, cells were washed with complete media and either assayed immediately, or returned to cell culture incubator for 24, 48 or 72 h. Intracellular ATP levels were measured using a luciferase-based assay. Data are represented as the calculated absolute levels of ATP (nmol ATP/mg protein). Data shown are the mean ± SEM, n=4.

Supplementary Table 3. The effect of Mito-ChM on intracellular ATP levels in MDA-MB-231 cells. Cells were treated with Mito-ChM (1-20 µM) for indicated periods of time. After treatment, cells were washed with complete media and either assayed immediately, or returned to cell culture incubator for 24, 48 or 72 h. Intracellular ATP levels were measured using a luciferase-based assay. Data are represented as the calculated absolute levels of ATP (nmol ATP/mg protein). Data shown are the mean ± SEM, n=4.

Supplementary Table 4. The effect of Mito-ChM on intracellular ATP levels in MCF-10A cells. Cells were treated with Mito-ChM (1-20 µM) for indicated periods of time. After treatment, cells were washed with complete media and either assayed immediately, or returned to cell culture incubator for 24, 48 or 72 h. Intracellular ATP levels were measured using a luciferase-based assay. Data are represented as the calculated absolute levels of ATP (nmol ATP/mg protein). Data shown are the mean ± SEM, n=4.

Supplementary Table 5. Effects of Mito-ChM on body weight and tissue weight in xenograft mouse models. Data shown are the mean ± SEM, (n=10, control group and n=9, Mito-ChM treated group)

Supplementary Table 1. Effects of Mito-ChM on ECAR, ATP-linked OCR and maximal OCR in MCF-7 and MCF-10A cells.

	4 h treatment					
	**ECAR**		**ATP-Linked OCR**		**Maximal OCR**	
	**MCF-7**	**MCF-10A**	**MCF-7**	**MCF-10A**	**MCF-7**	**MCF-10A**
**Control**	100.0 ± 8.7	100.0 ± 16.1	100.0 ± 7.3	100.0 ± 2.6	100.0 ± 1.8	100.0 ± 4.5
**Mito-ChM 1 μM**	228.9 ± 28.9*	327.5 ± 15.4	7.2 ± 4.6*	-6.6 ± 0.9	27.6 ± 3.4	31.5 ± 1.4
**Mito-ChM 3 μM**	189.0 ± 9.8**	274.9 ± 15.4	8.5 ± 4.8	9.1 ± 1.3	15.8 ± 4.7	10.0 ± 0.7
**Mito-ChM 10 μM**	45.3 ± 15.9**	257.3 ± 17.8	-1.7 ± 0.5*	2.8 ± 0.9	1.0 ± 0.7	0.3 ± 0.8

	4 h treatment with washout for 24 h					
	**ECAR**		**ATP-Linked OCR**		**Maximal OCR**	
	**MCF-7**	**MCF-10A**	**MCF-7**	**MCF-10A**	**MCF-7**	**MCF-10A**
**Control**	100.0 ± 5.8	100.0 ± 13.6	100.0 ± 9.1	100.0 ± 8.0	100.0 ± 9.0	100.0 ± 4.8
**Mito-ChM 1 μM**	138.9 ± 18.5	134.7 ± 21.2	69.1 ± 8.3	81.6 ± 5.7	81.4 ± 8.2	81.7 ± 6.4
**Mito-ChM 3 μM**	159.9 ± 11.3	163.8 ± 21.6	12.6 ± 3.1**	48.1 ± 1.3	39.3 ± 4.6	43.9 ± 1.7
**Mito-ChM 10 μM**	2.9 ± 1.2**	171.5 ± 18.4	-2.7 ± 2.2	2.7 ± 1.7	0.1 ± 0.2	2.0 ± 1.7

	4 h treatment with washout for 48 h					
	**ECAR**		**ATP-Linked OCR**		**Maximal OCR**	
	**MCF-7**	**MCF-10A**	**MCF-7**	**MCF-10A**	**MCF-7**	**MCF-10A**
**Control**	100.0 ± 10.3	100.0 ± 7.8	100.0 ± 7.8	100.0 ± 2.2	100.0 ± 8.8	100.0 ± 6.3
**Mito-ChM 1 μM**	106.8 ± 9.1	129.5 ± 15.1	58.5 ± 2.5**	116.0 ± 9.8	75.7 ± 3.4**	109.4 ± 5.6
**Mito-ChM 3 μM**	112.5 ± 5.4	141.4 ± 15.7	15.8 ± 2.8**	90.9 ± 1.2	48.0 ± 5.5**	95.1 ± 2.7
**Mito-ChM 10 μM**	0.8 ± 0.5**	138.6 ± 9.2	0.02 ± 0.03**	60.9 ± 3.9	0.05 ± 0.1**	56.4 ± 1.5

	4 h treatment with washout for 72 h					
	**ECAR**		**ATP-Linked OCR**		**Maximal OCR**	
	**MCF-7**	**MCF-10A**	**MCF-7**	**MCF-10A**	**MCF-7**	**MCF-10A**
**Control**	100.0 ± 6.2	100.0 ± 14.1	100.0 ± 2.0	100.0 ± 6.3	100.0 ± 2.0	100.0 ± 3.1
**Mito-ChM 1 μM**	116.8 ± 11.9	100.7 ± 6.3	53.7 ± 3.9**	116.1 ± 2.6	76.1 ± 5.1**	116.9 ± 4.8
**Mito-ChM 3 μM**	109.0 ± 19.8	101.4 ± 8.7	4.6 ± 3.1**	106.5 ± 5.9	26.9 ± 5.1**	99.6 ± 2.4
**Mito-ChM 10 μM**	0.7 ± 0.2**	143.5 ± 22.4	0.02 ± 0.02**	85.6 ± 7.4	0.01 ± 0.01**	79.4 ± 5.3

Supplementary Table 2. The effect of Mito-ChM on intracellular ATP levels in MCF-7 cells.

	Incubation time without washout				
**Mito-ChM** (μM)	***1 h***	***2 h***	***4 h***	***6 h***	***8 h***
**0**	25.6 ± 2.2	24.5 ± 1.8	26.6 ± 1.6	26.0 ± 1.7	26.1 ± 1.9
**1**	20.2 ± 2.0	19.8 ± 1.0	21.7 ± 1.0	23.3 ± 1.5	23.4 ± 1.5
**3**	23.3 ± 1.9	24.5 ± 1.7	27.6 ± 2.1	29.1 ± 2.1	27.0 ± 2.1
**5**	24.1 ± 1.8	27.1 ± 1.8	26.0 ± 2.3	28.3 ± 2.3	28.3 ± 1.8
**10**	25.7 ± 1.8	24.9 ± 2.0	27.7 ± 2.3	25.9 ± 2.9	24.2 ± 3.5
**15**	24.8 ± 1.8	25.8 ± 1.7	23.0 ± 3.0	19.6 ± 3.8	9.3 ± 2.0
**20**	22.5 ± 2.6	22.2 ± 2.9	18.2 ± 3.2	10.0 ± 2.5	3.1 ± 1.0
	Incubation time with washout for 24 h				
**Mito-ChM** (μM)	***1 h***	***2 h***	***4 h***	***6 h***	***8 h***
**0**	23.9 ± 0.6	24.8 ± 0.5	26.0 ± 0.9	25.6 ± 0.4	24.8 ± 0.7
**1**	23.2 ± 0.4	23.2 ± 0.7	23.3 ± 0.7	24.8 ± 0.4	24.7 ± 0.4
**3**	21.6 ± 1.0	22.2 ± 0.8	23.9 ± 0.8	22.3 ± 1.4	21.8 ± 1.4
**5**	19.7 ± 0.9	20.4 ± 1.4	24.1 ± 0.1	22.9 ± 0.4	24.5 ± 1.6
**10**	22.7 ± 0.6	22.1 ± 0.9	21.4 ± 0.6	18.5 ± 0.7	12.7 ± 0.6
**15**	21.7 ± 1.0	20.5 ± 1.1	12.4 ± 0.7	6.3 ± 1.1	1.1 ± 0.2
**20**	21.0 ± 0.5	14.5 ± 1.3	4.2 ± 0.5	0.7 ± 0.1	0.7 ± 0.1
	Incubation time with washout for 48 h				
**Mito-ChM** (μM)	***1 h***	***2 h***	***4 h***	***6 h***	***8 h***
**0**	21.7 ± 0.7	21.9 ± 0.9	22.3 ± 0.6	22.5 ± 0.7	22.7 ± 1.0
**1**	21.0 ± 0.8	20.8 ± 0.6	21.0 ± 0.6	21.6 ± 0.5	21.0 ± 0.4
**3**	18.4 ± 1.1	18.8 ± 0.6	18.3 ± 1.2	18.2 ± 0.7	18.1 ± 0.5
**5**	17.7 ± 0.6	17.9 ± 0.2	18.7 ± 0.7	18.8 ± 0.5	16.9 ± 1.2
**10**	18.4 ± 0.5	18.7 ± 0.3	14.2 ± 1.0	10.6 ± 0.8	4.9 ± 0.6
**15**	17.6 ± 1.0	13.1 ± 0.6	3.3 ± 0.2	0.9 ± 0.0	0.6 ± 0.0
**20**	14.0 ± 0.9	6.3 ± 1.1	0.6 ± 0.1	0.3 ± 0.0	0.5 ± 0.1
	Incubation time with washout for 72 h				
**Mito-ChM** (μM)	***1 h***	***2 h***	***4 h***	***6 h***	***8 h***
**0**	23.6 ± 0.4	23.7 ± 0.4	24.1 ± 0.6	20.2 ± 1.0	21.7 ± 0.9
**1**	20.6 ± 0.8	21.5 ± 0.9	20.8 ± 0.3	20.8 ± 0.4	19.3 ± 0.8
**3**	17.0 ± 0.5	16.2 ± 0.7	12.9 ± 0.6	13.9 ± 1.2	15.7 ± 0.6
**5**	14.4 ± 0.8	15.8 ± 0.6	14.4 ± 0.2	15.3 ± 0.8	15.0 ± 0.6
**10**	15.4 ± 0.7	12.1 ± 0.6	6.4 ± 0.8	4.2 ± 0.7	2.2 ± 0.5
**15**	11.4 ± 0.6	4.6 ± 0.8	0.6 ± 0.1	0.4 ± 0.0	0.4 ± 0.0
**20**	7.8 ± 0.9	1.9 ± 0.5	0.3 ± 0.0	0.3 ± 0.0	0.4 ± 0.1

Supplementary Table 3. The effect of Mito-ChM on intracellular ATP levels in MDA-MB-231 cells.

	Incubation time without washout				
**Mito-ChM** (µM)	***1 h***	***2 h***	***4 h***	***6 h***	***8 h***
**0**	36.2 ± 0.8	33.3 ± 3.2	36.3 ± 1.4	35.3 ± 1.2	35.9 ± 1.7
**1**	31.4 ± 2.1	35.0 ± 0.9	36.0 ± 0.9	34.5 ± 2.2	37.7 ± 0.4
**3**	36.0 ± 1.8	34.9 ± 0.9	34.0 ± 1.7	34.4 ± 1.1	38.7 ± 1.0
**5**	34.1 ± 0.7	30.0 ± 2.1	31.4 ± 2.8	37.2 ± 1.0	35.1 ± 2.9
**10**	33.8 ± 2.1	30.8 ± 1.3	34.4 ± 1.5	37.4 ± 0.5	36.4 ± 0.5
**15**	33.3 ± 2.0	35.1 ± 1.6	34.0 ± 1.6	32.3 ± 1.8	28.6 ± 0.9
**20**	33.5 ± 0.6	30.5 ± 3.6	30.0 ± 2.4	23.8 ± 2.2	9.5 ± 2.0
	Incubation time with washout for 24 h				
**Mito-ChM** (µM)	***1 h***	***2 h***	***4 h***	***6 h***	***8 h***
**0**	28.2 ± 1.6	29.7 ± 0.7	28.4 ± 0.8	28.7 ± 2.0	27.3 ± 1.0
**1**	28.1 ± 0.5	27.3 ± 0.5	29.9 ± 1.2	29.9 ± 0.5	27.2 ± 1.5
**3**	32.3 ± 0.8	30.8 ± 2.5	32.8 ± 2.4	34.0 ± 1.2	34.0 ± 0.4
**5**	32.7 ± 3.3	32.9 ± 0.1	27.9 ± 3.1	31.0 ± 0.6	30.0 ± 1.1
**10**	30.4 ± 0.8	27.3 ± 1.8	27.3 ± 1.0	26.1 ± 0.8	19.8 ± 1.1
**15**	29.0 ± 2.8	25.2 ± 2.2	18.8 ± 1.1	6.5 ± 1.0	1.5 ± 0.2
**20**	28.2 ± 1.1	23.8 ± 0.4	3.4 ± 0.6	0.9 ± 0.1	1.1 ± 0.1
	Incubation time with washout for 48 h				
**Mito-ChM** (µM)	***1 h***	***2 h***	***4 h***	***6 h***	***8 h***
**0**	26.9 ± 0.9	27.0 ± 0.6	26.0 ± 0.9	25.6 ± 1.1	26.6 ± 0.4
**1**	28.2 ± 0.4	28.2 ± 0.2	25.4 ± 2.1	25.7 ± 2.4	27.0 ± 0.7
**3**	22.5 ± 1.3	25.5 ± 0.4	24.1 ± 1.2	24.4 ± 0.5	24.6 ± 0.2
**5**	26.2 ± 0.3	26.7 ± 0.8	25.4 ± 0.9	25.6 ± 0.2	24.2 ± 0.5
**10**	25.0 ± 0.5	23.6 ± 0.8	21.3 ± 0.4	16.8 ± 0.3	13.1 ± 1.6
**15**	21.9 ± 0.2	18.5 ± 0.9	7.1 ± 1.3	0.8 ± 0.1	0.7 ± 0.0
**20**	20.4 ± 0.6	12.3 ± 0.7	0.7 ± 0.1	0.5 ± 0.1	0.6 ± 0.0
	Incubation time with washout for 72 h				
**Mito-ChM** (µM)	***1 h***	***2 h***	***4 h***	***6 h***	***8 h***
**0**	28.5 ± 0.8	27.6 ± 1.0	27.8 ± 1.3	28.8 ± 1.1	27.9 ± 0.7
**1**	27.9 ± 1.1	29.0 ± 0.7	27.5 ± 1.6	25.9 ± 1.0	27.2 ± 1.4
**3**	26.2 ± 0.9	26.3 ± 0.9	25.2 ± 1.6	25.5 ± 1.3	25.4 ± 0.8
**5**	24.0 ± 1.3	23.3 ± 1.6	24.3 ± 1.4	22.7 ± 0.9	19.9 ± 0.3
**10**	20.2 ± 1.0	18.5 ± 0.8	13.9 ± 1.5	10.5 ± 0.7	6.6 ± 0.5
**15**	15.6 ± 1.0	8.6 ± 0.4	1.3 ± 0.5	0.5 ± 0.0	0.6 ± 0.1
**20**	12.3 ± 0.5	2.5 ± 0.3	0.5 ± 0.0	0.4 ± 0.0	0.5 ± 0.1

Supplementary Table 4. The effect of Mito-ChM on intracellular ATP levels in MCF-10A cells.

	Incubation time without washout				
**Mito-ChM (μM)**	***1 h***	***2 h***	***4 h***	***6 h***	***8 h***
**0**	25.0 ± 0.5	24.6 ± 1.1	26.6 ± 0.5	24.6 ± 1.5	25.6 ± 1.0
**1**	20.3 ± 0.8	22.4 ± 0.6	26.2 ± 1.1	28.1 ± 1.5	31.6 ± 1.7
**3**	29.8 ± 0.2	29.9 ± 0.8	32.6 ± 1.4	33.9 ± 0.7	35.1 ± 1.1
**5**	29.1 ± 1.0	30.9 ± 0.2	30.9 ± 1.0	32.9 ± 0.7	32.5 ± 0.7
**10**	28.7 ± 1.0	29.9 ± 0.8	29.5 ± 1.4	28.6 ± 1.4	27.1 ± 1.7
**15**	27.9 ± 0.5	28.4 ± 1.4	27.0 ± 1.6	29.1 ± 0.3	28.9 ± 0.7
**20**	27.0 ± 1.2	29.8 ± 1.1	29.0 ± 0.5	27.7 ± 0.8	26.2 ± 0.6
	Incubation time with washout for 24 h				
**Mito-ChM (μM)**	***1 h***	***2 h***	***4 h***	***6 h***	***8 h***
**0**	27.4 ± 0.6	27.4 ± 0.7	26.9 ± 0.9	27.2 ± 0.7	27.3 ± 0.5
**1**	27.7 ± 0.5	27.3 ± 0.6	27.6 ± 0.7	26.9 ± 1.0	28.4 ± 1.0
**3**	31.2 ± 0.8	31.6 ± 0.7	32.1 ± 0.8	31.8 ± 0.4	31.7 ± 0.8
**5**	31.7 ± 1.2	33.3 ± 0.9	33.9 ± 0.8	32.4 ± 0.6	33.6 ± 1.0
**10**	34.4 ± 0.8	33.5 ± 1.0	33.4 ± 0.8	32.0 ± 1.3	32.6 ± 0.6
**15**	33.9 ± 1.8	34.5 ± 1.2	32.5 ± 0.9	32.8 ± 1.0	33.0 ± 0.8
**20**	33.8 ± 1.1	35.0 ± 1.1	33.4 ± 0.7	31.4 ± 0.8	32.2 ± 0.7
	Incubation time with washout for 48 h				
**Mito-ChM (μM)**	***1 h***	***2 h***	***4 h***	***6 h***	***8 h***
**0**	25.7 ± 0.2	25.2 ± 0.6	25.6 ± 0.4	26.8 ± 0.5	26.3 ± 0.4
**1**	28.3 ± 0.8	26.5 ± 0.8	27.1 ± 0.7	26.8 ± 0.5	27.1 ± 0.3
**3**	27.1 ± 0.8	26.3 ± 0.4	25.8 ± 0.3	25.9 ± 0.3	25.4 ± 0.4
**5**	24.7 ± 0.6	24.7 ± 0.9	24.9 ± 0.6	25.1 ± 0.7	25.3 ± 0.4
**10**	25.3 ± 0.6	26.1 ± 1.1	25.3 ± 0.9	24.4 ± 1.1	23.7 ± 0.9
**15**	27.6 ± 0.9	25.3 ± 0.9	21.9 ± 1.2	21.9 ± 1.4	20.6 ± 0.5
**20**	27.2 ± 1.0	23.7 ± 1.7	22.1 ± 0.9	20.8 ± 1.3	19.7 ± 1.0
	Incubation time with washout for 72 h				
**Mito-ChM (μM)**	***1 h***	***2 h***	***4 h***	***6 h***	***8 h***
**0**	29.9 ± 0.7	30.7 ± 0.4	30.4 ± 1.0	30.3 ± 0.8	30.6 ± 0.2
**1**	30.8 ± 0.7	30.4 ± 0.8	30.1 ± 0.4	30.0 ± 0.2	29.2 ± 0.4
**3**	30.3 ± 0.9	30.0 ± 0.7	30.0 ± 0.6	29.4 ± 0.5	27.8 ± 0.9
**5**	29.5 ± 0.5	30.1 ± 0.4	29.6 ± 0.3	28.7 ± 1.2	27.8 ± 0.3
**10**	27.9 ± 0.6	27.9 ± 1.2	28.2 ± 0.5	27.8 ± 0.3	25.1 ± 0.7
**15**	28.2 ± 0.9	29.4 ± 0.7	27.6 ± 0.7	24.3 ± 1.3	21.3 ± 0.2
**20**	29.5 ± 1.8	29.3 ± 0.7	25.6 ± 0.8	23.0 ± 0.2	18.6 ± 0.7

Supplementary Table 5. Effects of Mito-ChM on body weight and tissue weight in xenograft mouse models. Data shown are the mean ± SEM, (n=10, control group and n=9, Mito-ChM treated group)

	Body weight (g)	Kidney (mg)	Liver (mg)	Heart (mg)
**Control**	21.7 ± 0.4	148.6 ± 4.3	1163.8 ± 50.2	136.1 ± 6.2
**Mito-ChM (60 mg/kg)**	19.1 ± 0.7*	140.6 ± 5.3	1022.4 ± 53.1	123.2 ± 6.6
